# Silver-based bimetallic nanozyme fabrics with peroxidase-mimic activity for urinary glucose detection

**DOI:** 10.1007/s00216-024-05483-7

**Published:** 2024-08-17

**Authors:** Sanjana Naveen Prasad, Sanje Mahasivam, Rajesh Ramanathan, Vipul Bansal

**Affiliations:** Sir Ian Potter NanoBioSensing Facility, NanoBiotechnology Research Laboratory (NBRL), School of Science, RMIT University, Melbourne, VIC 3000 Australia

**Keywords:** Bimetallic nanoparticles, Functional fabrics, Nanozyme, Colorimetric, Atypical peroxidase, Urinalysis

## Abstract

The enhanced catalytic properties of bimetallic nanoparticles have been extensively investigated. In this study, bimetallic Ag-M (M = Au, Pt, or Pd) cotton fabrics were fabricated using a combination of electroless deposition and galvanic replacement reactions, and improvement in their peroxidase-mimicking catalytic activity compared to that of the parent Ag fabric was studied. The Ag-Pt bimetallic nanozyme fabric, which showed the highest catalytic activity and ability to simultaneously generate hydroxyl (•OH) and superoxide (O_2_^•−^) radicals, was assessed as a urine glucose sensor. This nanozyme fabric sensor could directly detect urinary glucose in the pathophysiologically relevant high millimolar range without requiring sample predilution. The sensor could achieve performance on par with that of the current clinical gold standard assay. These features of the Ag-Pt nanozyme sensor, particularly its ability to avoid interference effects from complex urinary matrices, position it as a viable candidate for point-of-care urinary glucose monitoring.

**Graphical Abstract:**

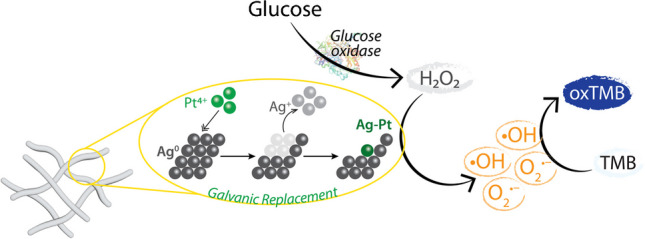

**Supplementary Information:**

The online version contains supplementary material available at 10.1007/s00216-024-05483-7.

## Introduction

Bimetallic and multi-metallic nanomaterials have attracted considerable attention in the field of catalysis because of their superior properties resulting from the synergistic or additive effects of their components [1]. The synthesis routes used to fabricate these nanomaterials allow the control of their size, shape, and morphology, and thereby their properties [2]. Some commonly used synthesis approaches include co-reduction [1], thermal decomposition [1], microwave [3], seeded growth [4], and galvanic replacement (GR) [5–11]. Among these, GR approaches offer the simplicity of a facile, single-step reaction to convert a single metal into a bimetallic system without requiring an externally applied potential [9]. GR reactions typically involve the exchange of atoms driven by the difference in the standard reduction potential between a sacrificial metal template and a different metal ion in the solution [7, 8]. This approach has become a versatile tool for the fabrication of bimetallic nanomaterials because the morphology and composition of the final nanostructure can be precisely controlled by factors such as the metal ion concentration, their oxidation state, template morphology, and reaction medium [2, 5–9, 12–15]. A key benefit of incorporating a second metal is that it can expand the structural diversity (including Janus, core–shell, alloy, and intermetallics), spatial ordering, and atomic distribution, all of which can influence the overall catalytic performance [16]. Further, the addition of a small amount of catalytically active yet expensive noble metals such as gold, platinum, and palladium to a low-cost template metal such as silver or copper via GR reactions can greatly increase the catalytic turnover frequency, enhancing the potential of these systems for a myriad of practical applications [17]. These include the use of bimetallic nanomaterials with improved catalytic efficiencies for solar photocatalysis [15, 18], light-induced reductive catalysis [10, 19], and enhanced photothermal activity [20].

Another area where bimetallic nanomaterials have demonstrated potential is their ability to promote enzyme-mimicking biocatalysis reactions, commonly referred to as nanozyme reactions. Nanozymes have been used in applications such as sensing, microbial control, pollution control, environmental remediation, diagnostics, and prodrug therapies [17, 21–38]. While most reported nanozymes mimic the catalytic activity of oxidoreductases such as peroxidase and oxidase enzymes, new nanozymes are reported for activities such as catalase, superoxide dismutase, laccase, and hydrolases [25, 38].

The significance of bimetallic nanozymes has been realised recently, with reports ranging from improved detection limits to multi-enzyme-mimic behaviour beginning to emerge. An appealing prospect of multi-metallic nanomaterials is their potential to simultaneously generate more than one reactive oxygen species (ROS), the key mechanism through which most nanozymes tend to operate. These include a Au/Mo nanohybrid that exhibited superior in situ photogeneration of singlet oxygen (^1^O_2_) and •OH radicals, [39] and a Pt/Fe simultaneously generating •OH and superoxide (O_2_^•−^) radicals [40].

Taking advantage of these additive and/or synergistic effects of bimetallic systems, the current work focuses on combining two noble metals (Ag and one other metal) to create bimetallic nanozyme fabrics. The high catalytic activity of this Ag-Pt bimetallic system allowed direct detection of urinary glucose in the millimolar range, as opposed to the micromolar range mostly achieved by single metal nanozymes due to their limited catalytic activity. While most sensor technologies focus on achieving the lowest possible detection limit, where physiological analyte concentration is higher, it is preferable to develop sensor technologies that can directly detect the higher analyte concentration without sample predilution. Such is the case with physiological glucose concentration, as the biologically relevant concentration range for diabetes monitoring in urine is over 0.8 mM [41]. Another strategy to allow direct detection of a high analyte concentration is by loading a high amount of the nanozyme on a suitable template. This strategy overcomes the limitation of colloidal nanozymes whose sensor response saturates at higher analyte concentrations. By loading a high density of Ag nanozyme on cotton textiles, we could previously extend the operating range of the urinary glucose sensor to 2 mM, beyond which the sensor response saturated [22]. However, the human urinary glucose concentration in diabetic individuals and those with nephropathy and glucosuria could be as high as 14 mM [42]. Further, pets, such as cats, can also develop diabetes, and their urinary glucose concentration ranges between 5 and 50 mM [43, 44]. Therefore, to directly detect such high concentrations of urinary glucose without the need for sample predilution, nanozymes with higher catalytic activity are required.

Motivated by the above rationale, the current study combines the above two concepts wherein bimetallic nanozyme fabrics were created for the direct detection of urinary glucose. Specifically, Ag was combined with other noble metals, such as Au, Pt, or Pd, using a GR approach to create bimetallic nanozyme fabrics, and their catalytic activity was assessed. The most promising Ag-Pt nanozyme fabric, with a peroxidase-mimic activity was then utilised in combination with a colorimetric substrate 3,3′,5,5′-tetramethylbenzidine (TMB), to monitor glucose concentration in undiluted human urine. The outcomes of this study show the promise of the presented technology as a potential candidate for point-of-care urinary glucose monitoring.

## Experimental methodology

### Material synthesis, characterisation, and assessment of enzyme-mimic catalytic activities

Details of the materials and methods are provided in the Electronic Supplementary Material. Briefly, Ag nanoparticles were synthesised on cotton fabrics using an electroless deposition strategy, followed by a GR reaction to convert Ag into bimetallic nanoparticles, as detailed in our previous reports [10, 22]. The nanomaterials were characterised using a suite of microscopy and spectroscopy tools. These bimetallic nanoparticles on cotton fabrics were evaluated for their peroxidase-mimicking catalytic activity using chromogenic substrates including TMB, o-phenylenediamine dihydrochloride (OPD), and 2,2′-azino-bis(3-ethylbenzothiazoline-6-sulphonic acid) (ABTS). The optimal parameters were then determined, and the mechanism of the catalytic activity was assessed by probing the production of different ROS using relevant dyes. The steady-state kinetic parameters such as *K*_*m*_, the Michaelis constant, and *V*_*max*_, the maximum reaction velocity, were determined to understand the suitability of the Ag-Pt bimetallic nanozyme for the detection of glucose in urine.

### Colorimetric detection of glucose using Ag-Pt nanozyme fabric

The detection of glucose using the peroxidase-mimicking catalytic activity of the Ag-Pt fabric was carried out in two steps. In step one, 50 µL of varying glucose concentrations and 50 µL of glucose oxidase (GOx, 2 mg mL^−1^) were incubated at 37 °C for 30 min in 380 µL 50 mM sodium acetate buffer (pH 5). Next, 20 µL of TMB (5 mM) and Ag-Pt fabric (2 mg) were added to the reaction mixture and further incubated for 15 min, following which, the colorimetric response was measured at a wavelength of 652 nm. The dynamic range of glucose detection by the Ag-Pt fabric in buffer was obtained by plotting glucose concentration *vs* absorbance_652 nm_, followed by linear regression analysis. The limit of detection (LoD) was calculated using the Eq. *3.3* × *(standard error of the y-intercept/slope)* and the limit of quantification (LoQ) was calculated using the Eq. *10* × *(standard error of the y-intercept/slope)*. The accuracy of the sensor was determined using *(n/N)* × *100*, where *n* is the number of sensing events that fall within the target response and *N* is the total number of test events. The % precision was calculated by the coefficient of variation (CoV) method using the formula % precision = 100 – %CoV. To further evaluate the reproducibility of the nanozyme sensor, in-batch, intra-batch, and inter-batch accuracy and precision parameters were also determined. For determining the in-batch parameters, a single nanozyme fabric (post synthesis) was cut into multiple pieces post-synthesis before use in sensing (15 replicates). The intra-batch experiments involved the comparison of multiple nanozyme fabrics synthesised simultaneously in a single batch but performing the sensing assay on different days. Lastly, inter-batch variability was assessed by performing the assay using Ag-Pt fabrics prepared in separate batches. For these in-batch, inter-batch, and intra-batch studies, three glucose concentrations (0.1, 1, and 12 mM) were tested. The specificity of the sensor was also assessed by exposing it to a fixed concentration (10 mM) of glucose analogues, such as fructose, galactose, lactose, sucrose, and maltose, both independently and in combination with glucose (10 mM).

To detect glucose in complex biological fluids, urine samples were collected from five volunteers and stored in a refrigerator until further use. Glucose detection in urine samples was performed using two approaches:

- **Method 1—GOx-HRP**: 50 µL of the urine sample (10X dilution) and 50 µL of GOx (2 mg mL^−1^) were incubated in 360 µL of 50 mM sodium acetate buffer (pH 5) at 37 °C for 30 min. This was followed by the addition of 20 µL of TMB (5 mM) and 20 µL of HRP (1 µg mL^−1^ or 0.125 units) and a further incubation for 15 min. The colorimetric response from the reaction solution was measured at a wavelength of 652 nm.
- **Method 2—Ag-Pt fabric**: 50 µL of urine sample (undiluted) was added to 50 µL of GOx (2 mg mL^−1^) and incubated at 37 °C for 30 min in 380 µL of 50 mM sodium acetate buffer (pH 5). This was followed by adding 20 µL of TMB (5 mM) and Ag-Pt nanozyme fabric (2 mg) to the reaction mixture. The colorimetric responses at 652 nm were measured after 15 min of reaction.

A urine glucose calibration curve was first created. To do this, the urine sample from a healthy volunteer was first quantified using the laboratory gold standard GOx-HRP assay (Method 1) to determine the inherent glucose concentration. This assay resulted in no colour generation, suggesting that the urine sample contained undetectable amounts of glucose. Therefore, this sample was assumed to contain 0 mM of glucose. Subsequently, known amounts of glucose (powder form, mg) were directly dissolved in the undiluted urine sample to achieve a glucose concentration of 1–120 mM. These glucose-spiked urine samples were introduced to the Ag-Pt nanozyme sensor (Method 2), and the sensor response was calculated as a function of the relative increase in absorbance (%) and then fitted using a Hill function (OriginPro 2016). Further, the glucose concentrations in these spiked urine samples were determined using the laboratory gold standard (Method 1) and Ag-Pt nanozyme fabrics (Method 2) separately to compare their performances in terms of % recovery. Lastly, the same assay was conducted on urine samples of diabetic and healthy volunteers' to determine the glucose concentration.

## Results and discussion

### Synthesis of bimetallic Ag-M nanozyme fabrics (M = Au, Pd, or Pt)

**Scheme 1:**
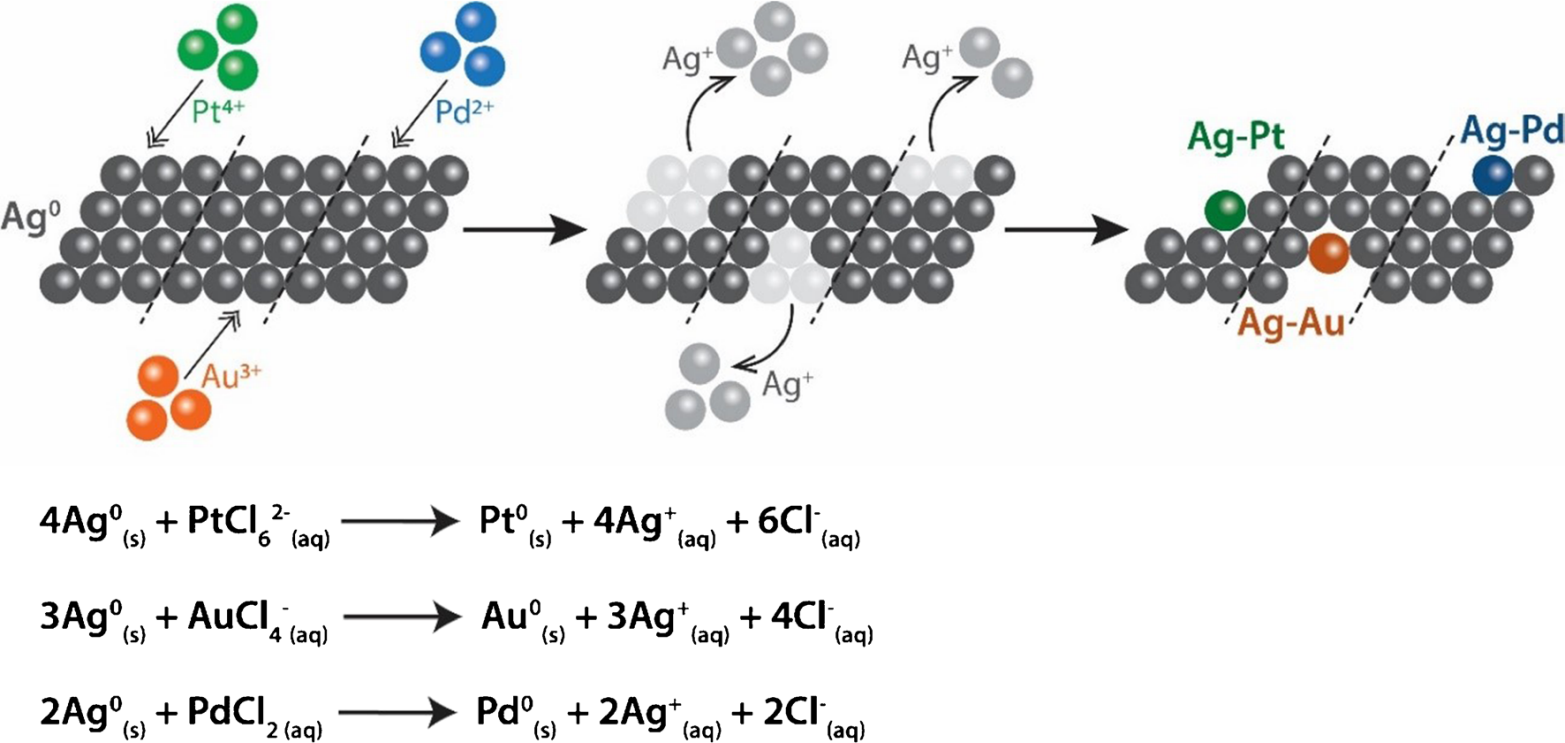
Schematic showing the production of bimetallic Ag-M nanozyme fabrics via galvanic replacement reactions between the Ag fabric and [AuCl_4_]^−^, Pd^2+^, or [PtCl_6_]^2−^ ions

The bimetallic Ag-M (M = Au, Pd, or Pt) nanozyme fabrics were synthesised by first creating the parent Ag fabric via an electroless deposition technique, as described in our previous work [22, 45]. The method involved sensitisation of cotton fabric in an acidic solution using tin chloride, followed by seeded growth of Pd^0^ nuclei, which acted as a catalyst for the subsequent deposition of silver nanoparticles onto the fabric via reduction of the diamine silver (I) complex [45]. These Ag fabrics were converted into bimetallic Ag-M fabrics by exposing the 1 × 1 cm^2^ Ag fabric to aqueous solutions of HAuCl_4_, PdCl_2_, or H_2_PtCl_6_. The favourable difference in the reduction potential between Ag^0^ (Ag^+^/Ag^0^ 0.799 V vs standard hydrogen electrode—SHE) and the metal ions (AuCl_4_^−^/Au^0^ 0.93 V vs SHE; Pd^2+^/Pd^0^ 0.95 V vs SHE; PtCl_6_^2−^/Pt^0^ 0.74 V vs SHE) initiated the spontaneous GR reactions between Ag and the respective metal ions (Scheme 1) [9]. This resulted in the oxidative dissolution of zerovalent Ag to Ag^+^ ions in the solution, and simultaneous reductive deposition of Au^0^, Pd^0^, or Pt^0^ from [AuCl_4_]^−^, Pd^2+^, or [PtCl_6_]^2−^ ions, respectively, onto Ag fabrics. The bimetallic Ag-Au, Ag-Pd, and Ag-Pt nanozyme fabrics produced using this method contained 0.13–0.18 mg of metal content per 2 mg of these fabrics, as determined using atomic emission spectroscopy (AES) (Fig. S1).

### Characterisation of Ag-M nanozyme fabrics

**Fig. 1:**
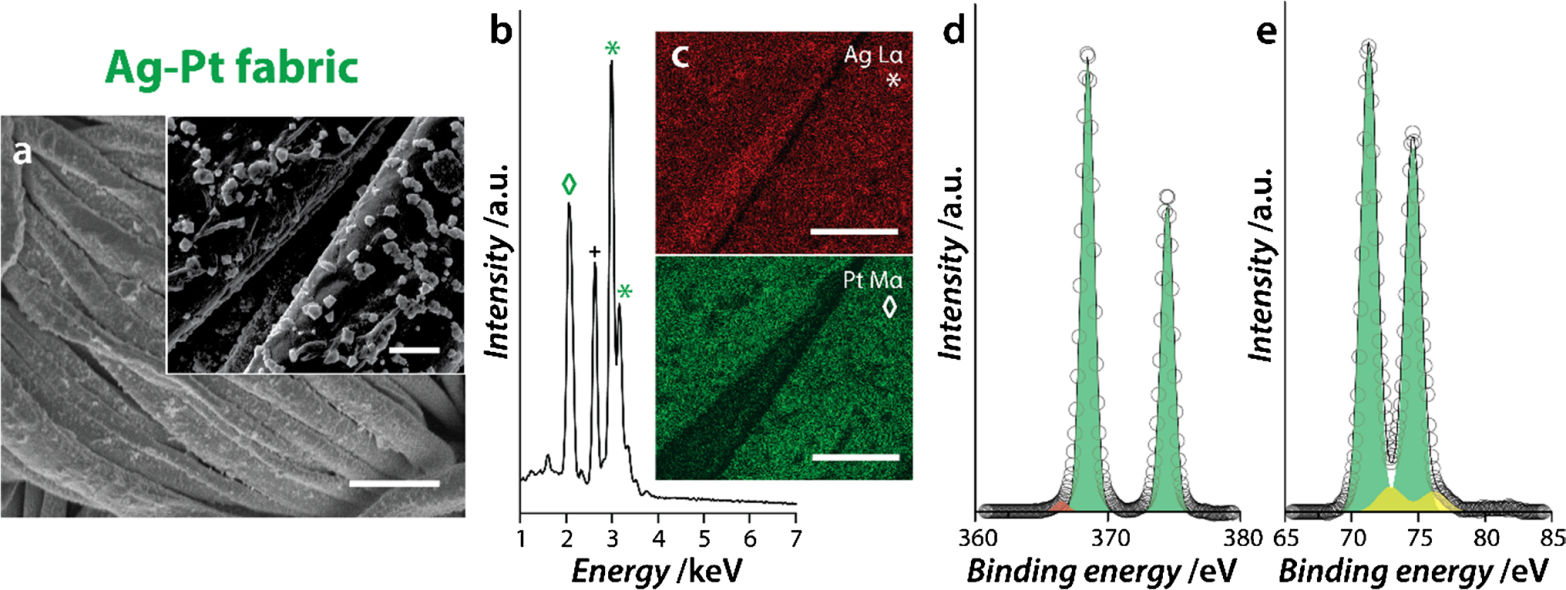
Characterisation of the Ag-Pt fabric, including **a** SEM images (scale bars correspond to 50 μm for the main figure and 5 μm for the inset); **b** EDX spectra where the asterisk represents Ag, the diamond symbol represents Pt, and the plus sign represents residual Cl; **c** EDX maps showing the distribution of the different metals (scale bars correspond to 10 μm); **d** Ag 3d XPS core level spectra and **e** Pt 4f XPS core level spectra

Scanning electron microscopy (SEM) images of the Ag-M fabrics clearly show the deposition of nanoparticles on the individual threads of the cotton fabrics (Fig. 1a showing Ag-Pt and Fig. S3a showing Ag-Au and Ag-Pd). The parent Ag fabric showed quasi-spherical Ag nanoparticles of sub-100 nm in size, as shown in our previous study (Fig. S2) [22]. SEM images obtained after the GR reactions revealed an increase in surface roughness due to the deposition of Au, Pd, or Pt metal (detailed SEM, EDX, and XPS analysis of Ag-Au and Ag-Pd fabrics is given in Electronic Supplementary Material (S3.2)). Although the overall particle size remained within 100 nm, clusters of nanoparticles were present on the surfaces of the individual threads of the cotton fabric (Fig. 1a inset and Fig. S3 insets). Energy-dispersive X-ray (EDX) spectral analysis showed characteristic energy lines associated with Ag Lα (2.98 keV and 3.15 keV) and from the additional metal in the bimetallic nanostructures. For instance, the Ag-Pt fabric showed a Pt Mα line at 2.05 keV (Fig. 1b), while Au Mα and Pd Lα lines were observed for Ag-Au and Ag-Pd fabrics, respectively (Fig S3b). Further, EDX elemental maps also indicated the uniform distribution of the bimetallic nanostructures across the surface of the fabric (Fig. 1c and Fig. S3c).

The oxidation states of the metals on the Ag-M fabrics were analysed using X-ray photoemission spectroscopy (XPS). All core level spectra were background-corrected and their binding energies (BEs) were aligned to the adventitious C 1*s* BE of 285 eV. The core level Ag 3*d* spectrum from all Ag-M fabrics revealed two characteristic core level splitting components, 3*d*_*5/2*_ and 3*d*_*3/2*_ (spin–orbit splitting of ~ 6 eV) with a 3*d*_*5/2*_ binding energy of 368.5 ± 0.1 eV [46], corresponding to Ag in the zerovalent oxidation state (Fig. 1d and Fig. S3d). A minor peak observed at low binding energy is due to the plasmon loss feature of the Ag nanoparticles [47]. XPS analysis of the Ag-Pt fabric revealed two Pt 4*f*_*7/2*_ components at ~ 71.3 eV and 72.9 eV corresponding to Pt^0^ and Pt^2+^, respectively (spin–orbit splitting ~ 3.3 eV between Pt 4*f*_*7/2*_ and 4*f*_*5/2*_)) [48] (Fig. 1e). Similarly, Au 4*f*_*5/2*_ and Au 4*f*_*3/2*_ splitting components were observed in Ag-Au fabric and Pd 3*d*_*5/2*_ and Pd 3*d*_*3/2*_ splitting components were observed in Ag-Pd fabrics. Overall, XPS analysis revealed that the Ag-M fabrics predominantly contained metallic forms of Ag and M (Fig. S3e), with minor ionic impurities in the case of Pd and Pt.

### Enzyme-mimicking catalytic activity of Ag and Ag-M nanozyme fabrics

TMB, a chromogenic substrate, can be oxidised to a blue charge transfer complex (*λ*_max_ = 652 nm) by losing one electron by natural peroxidases, oxidases, and their enzyme mimics [22, 49]. We utilised this property of TMB to compare the peroxidase and oxidase-mimicking catalytic activities of the Ag-M nanozyme fabrics with those of the Ag fabric [49]. In the absence of H_2_O_2_, the Ag-M fabrics revealed oxidase-mimicking catalytic activity (Fig. S4a). However, the oxidase-mimicking activities were poor, as TMB oxidation occurred gradually over time. The observed oxidase activity trend among the different nanozyme fabrics was Ag-Pd > Ag > Ag-Au > Ag-Pt. In contrast, when the catalytic activity was assessed in the presence of H_2_O_2_, the Ag fabric and the three Ag-M fabrics displayed high peroxidase-mimicking catalytic activities. This was reflected by the formation of a visible blue product within 2–4 min of the reaction. The superior peroxidase-mimicking activity of these nanozymes is reflected from 1.1, 1.5, and 2.8 orders of magnitude greater peroxidase activity of Ag-Pd, Ag-Au, and Ag-Pt than their respective oxidase activities when compared after 2 min of reaction. The three bimetallic nanozyme fabrics showed higher peroxidase-mimic activities than the parent Ag fabric, where the highest activity was observed for the Ag-Pt nanozyme fabric, which facilitated over two times higher amount of TMB oxidation than the Ag nanozyme within 2 min. After this initial surge, the concentration of the blue product decreased over time. This can be attributed to further oxidation of the blue TMB charge transfer complex into a yellow diimine derivative (*λ*_max_ = 450 nm), as evident in Fig. S4b. While most natural enzymes and nanozymes allow only the first stage of oxidation, leading to a blue product, double-oxidised TMB can also be directly produced by certain highly efficient nanozyme catalysts [17, 24].

**Fig. 2:**
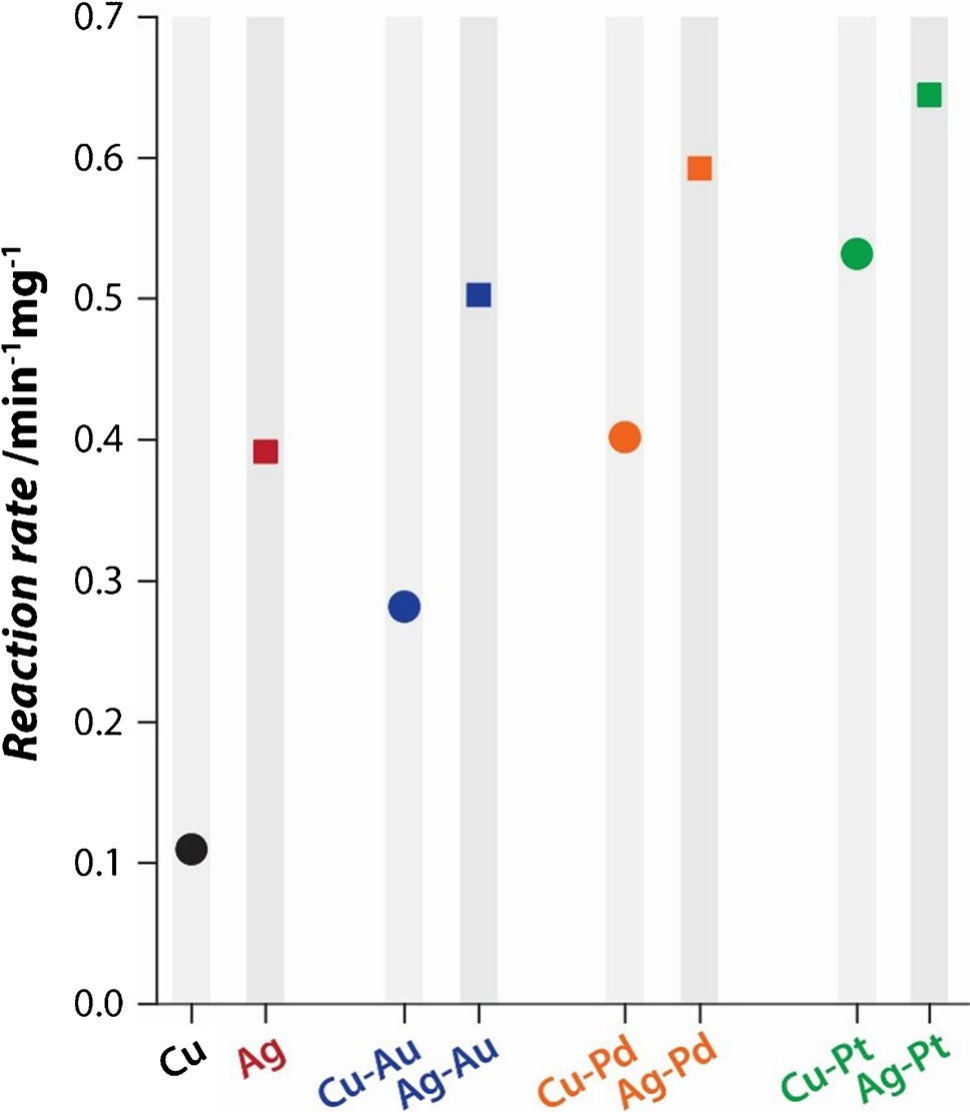
Comparison of the TMB oxidation rates (colourless to blue product) achieved by different nanozyme fabrics after normalising to equivalent weights of the active catalyst (metal) present on these fabrics. The reaction rates for the Cu and Cu-M fabrics are also plotted for comparison, and these were obtained from our previous study that employed similar reaction conditions [17]

Since the peroxidase-mimicking catalytic activity of the Ag-M fabrics was very high, which led to saturation of reactions within 2 min, the weight of the Ag-M fabrics was reduced to 1 mg to determine the rate of TMB oxidation by different nanozyme fabrics. Time-dependent TMB oxidation by these smaller-sized fabrics showed a consistent increase in activity over 20 min (Fig. S5a). This allowed the calculation of TMB oxidation rates by plotting *ln*(*A*_t_/*A*_0_) *vs*. time (where *A*_t_ is the absorbance at time *t* and *A*_0_ is the absorbance at 0 min) (Fig. S5b) and deriving the reaction rates from the linear regions of the curves. Further, the amount of active catalyst (total metal weight) varied across the different Ag-M fabrics (Fig. S1). To assess the relative catalytic activity of the nanozyme fabrics, the reaction rates were normalised to the equivalent metal mass in each sample (Fig. 2). The Ag-Pt nanozyme fabric showed 1.6 times higher catalytic activity than Ag fabric in forming the blue charge transfer complex. The order of catalytic activity of the nanozyme fabrics can be summarised as Ag-Pt > Ag-Pd > Ag-Au > Ag. A comparison of the catalytic activity of Ag-based nanozyme fabrics towards TMB oxidation with those of Cu-based fabrics previously reported by our team [17] shows that Ag-based fabrics offer superior nanozyme activity (data for Cu-based fabrics are also plotted in Fig. 2 for comparison). Interestingly, across both the Ag- and Cu-based hybrid fabric systems, the influence of additional metal on the overall nanozyme activity followed the trend of Pt > Pd > Au. A comparison of the two best-performing nanozymes from the Cu and Ag systems shows that the Ag-Pt fabric offers 1.2 times higher catalytic performance than Cu-Pt in producing the blue charge transfer product. Platinum is in fact considered one of the best catalysts for a range of industrially important chemical transformation reactions, and the trends of catalytic activities observed for TMB oxidation in our studies correlate well with the relative activities of these noble metals in promoting other catalytic reactions [16]. Regarding the Ag-Pt nanozyme fabric, it is also noteworthy that while its peroxidase-mimicking activity is the highest, it also has nearly negligible and the lowest oxidase-mimicking activity across all nanozyme fabrics (Fig. S4a). The development of a nanozyme-based glucose sensor requires the use of a glucose oxidase (GOx) enzyme to provide selectivity towards glucose. A nanozyme with high oxidase-mimic activity can non-specifically oxidise other analytes present in clinical samples (e.g., ascorbate, cholesterol, etc.), thus compromising the selectivity and accuracy of the sensor. In this context, the Ag-Pt nanozyme fabric was identified as the most promising candidate for the development of a urinary glucose sensor and was chosen for subsequent studies.

The leached metal ions did not promote the catalytic reaction suggesting that the peroxidase-mimicking catalytic activity was intrinsic to Ag-Pt fabric. Optimisation of the assay parameters further provided information about the reaction conditions required for the development of a glucose sensing platform based on the Ag-Pt nanozyme (detailed discussion in the Electronic Supplementary Material (S5)). To summarise, the optimum parameters for Ag-Pt nanozyme fabric were 37 °C and pH 5 while using TMB as the substrate. This was followed by the evaluation of the catalytic activity in terms of the steady-state kinetic parameters—Michaelis constant (*K*_*m*_) and maximum initial velocity of reaction (*V*_*max*_). The outcomes showed that the Ag-Pt nanozyme had a high affinity to H_2_O_2_ (~ 1.8 mM) and TMB (~ 0.2 mM). A detailed discussion of the experimental results is in the Electronic Supplementary Material (S6.2). Further, the underlying mechanism of the peroxidase-mimicking catalytic activity was determined by investigating the generation of reactive oxygen species (ROS) including hydroxyl radicals (•OH), superoxide radicals (O_2_^•−^), and singlet oxygen (^1^O_2_), in the presence of H_2_O_2_. Overall, the Ag-Pt nanozyme fabric could facilitate the decomposition of H_2_O_2_ via the simultaneous production of two ROS, •OH and O_2_^•−^ radicals through a series of reactions that could be explained by the classical Haber–Weiss mechanism [50, 51]. Although •OH radicals are widely reported in nanozyme-mediated peroxidase-mimic reactions, the production of O_2_^•−^ radicals involved a free radical chain reaction where the efficiency of the O_2_^•−^ production depended on the oxidation potential of the catalyst. Based on previous studies, the highest rate constant for this reaction was observed for Ag, followed by Pt [52]. Therefore, the production of both •OH and O_2_^•−^ radicals was feasible using Ag-Pt nanozyme. A detailed explanation of the possible mechanism by which multiple ROS species are produced by the Ag-Pt fabric is provided in the Electronic Supplementary Material (S7.2).

### Glucose sensing

Having determined the optimum assay conditions and the mechanism by which the Ag-Pt nanozyme catalyses the oxidation of peroxidase substrates, the outstanding peroxidase-mimic ability of the Ag-Pt nanozyme was utilised to develop a colorimetric sensor for the detection of urinary glucose. This was achieved by employing glucose oxidase (GOx), a natural enzyme that selectively oxidises glucose to gluconic acid, even in the presence of other (bio)molecules [53]. A by-product of this reaction is H_2_O_2_ which can then participate in the peroxidase-mimicking reaction catalysed by Ag-Pt nanozyme, subsequently leading to an indirect quantification of glucose concentration. Typically, in reactions involving both a nanozyme and GOx, glucose is first incubated with GOx at pH 7 to promote enzymatic oxidation of glucose, followed by changing the conditions to achieve optimal nanozyme activity (pH 4 or 5) [22, 54, 55]. In the present study, the GOx enzyme from *Aspergillus niger*, which has optimal enzymatic activity at pH 5, was used [56]. As the Ag-Pt nanozyme also offers optimal catalytic performance at pH 5 (Fig. S7a), the assay could be simplified to a single buffer system.

**Fig. 3:**
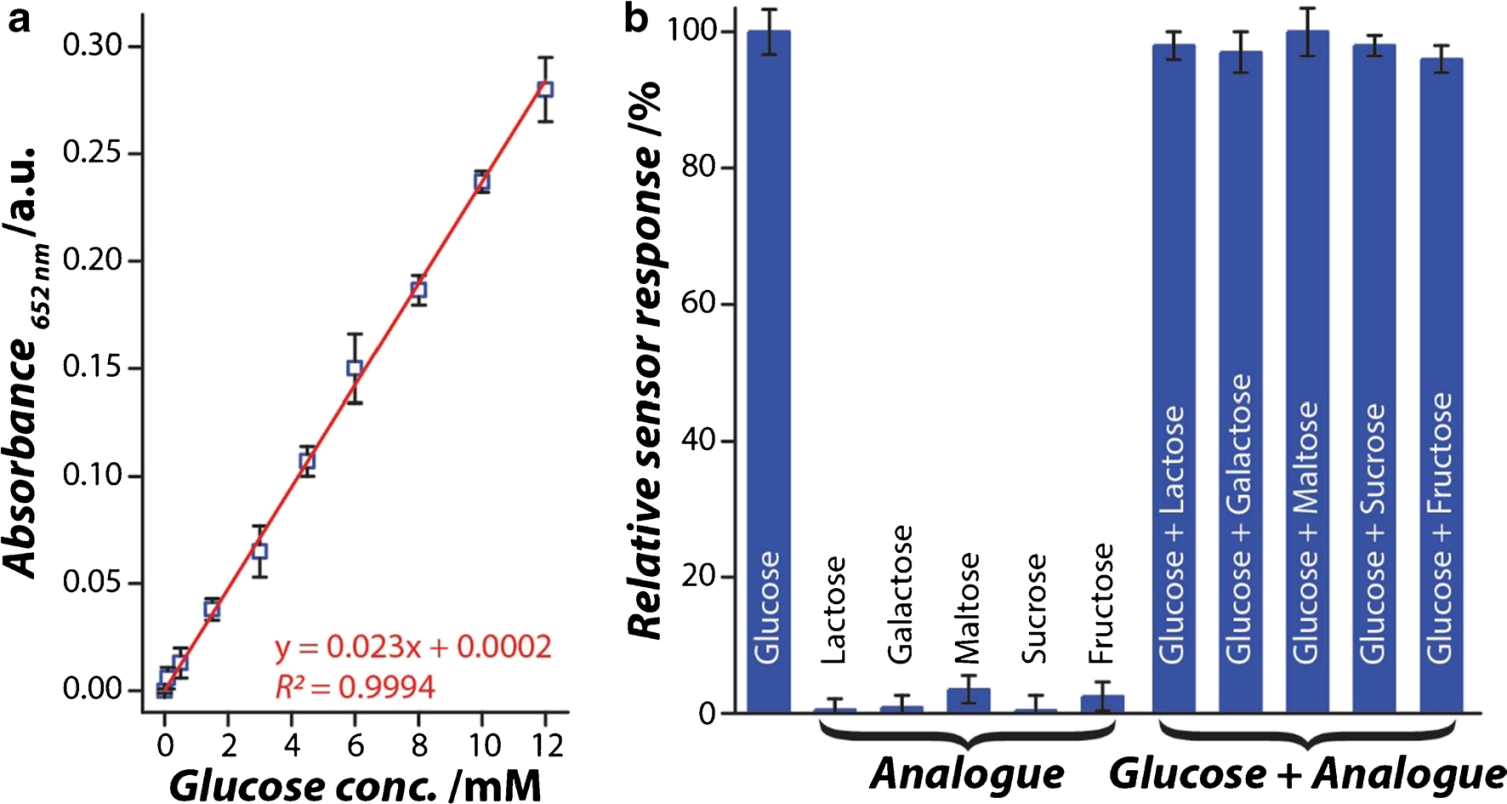
Glucose sensing capabilities of Ag-Pt nanozyme fabric. **a** Linear absorbance response to increasing glucose concentration, and **b** sensor specificity for glucose detection, where the concentration of glucose and its analogues was 10 mM

To determine the dynamic range of the nanozyme sensor, fixed concentrations of GOx and Ag-Pt nanozyme fabrics were exposed to increasing glucose concentrations (0–20 mM). Although the sensor developed a colorimetric response over the entire range of glucose concentrations, a linear response was observed only between 0.1 and 12 mM (Fig. 3a). The limit of detection (LoD) calculated from the slope and standard error of the Y-intercept (details in “Colorimetric detection of glucose using Ag-Pt nanozyme fabric”) showed that the sensor could potentially detect glucose concentrations as low as 0.06 mM (with the limit of quantification (LoQ) being 0.18 mM), whereas the precision and accuracy were calculated to be 96.5% and 93.3% (at 5% contingency), respectively. Sensor specificity was evaluated by exposing the sensor to glucose analogues, including fructose, galactose, sucrose, lactose, and maltose, independently and in combination with glucose (Fig. 3b). In both cases, the analogues showed minimal interference with the quantification of glucose concentration, whereas the sensor showed a < 5% response to the analogues independently. This high specificity of the sensor is expected, as the glucose oxidase enzyme acts as the recognition element in the current sensing platform and shows a high propensity to specifically oxidise glucose despite the presence of glucose analogues.

We also compared the glucose sensing performance of the Ag-Pt nanozyme with other fabric-based glucose sensing platforms, including monometallic Ag fabric [22], Cu nanozyme [24], and the bimetallic Cu-Pt nanozyme [17] (Table S2). In comparison to the Ag fabric nanozyme sensor, the detection range of all other systems showed the ability to detect glucose in the physiological range with some compromise in the accuracy (5% contingency). Comparing the two bimetallic fabrics viz. Cu-Pt nanozyme and Ag-Pt nanozyme, the limit of detection of the Ag-Pt nanozyme was ~ 14 times lower than the Cu-Pt fabric without compromising the accuracy and precision.

Further, the sensor reproducibility was assessed by exposing the Ag-Pt fabric nanozyme to three independent concentrations of glucose—0.1 mM, 1 mM, and 12 mM (Table S3). First, the glucose sensing assay was conducted 15 times using Ag-Pt fabrics prepared in a single batch (in-batch). Next, the intra-batch variation was determined by conducting the glucose sensing assay on different days using the Ag-Pt fabrics prepared in a single batch. Lastly, the glucose sensing assay was performed using the Ag-Pt fabrics prepared in separate batches. Irrespective of using the Ag-Pt nanozyme from a single or different batch, the precision was > 94% (*n* = 15) while the sensor accuracy was between 91 and 100% (at 5% contingency) or 100% (at 10% contingency).

**Fig. 4:**
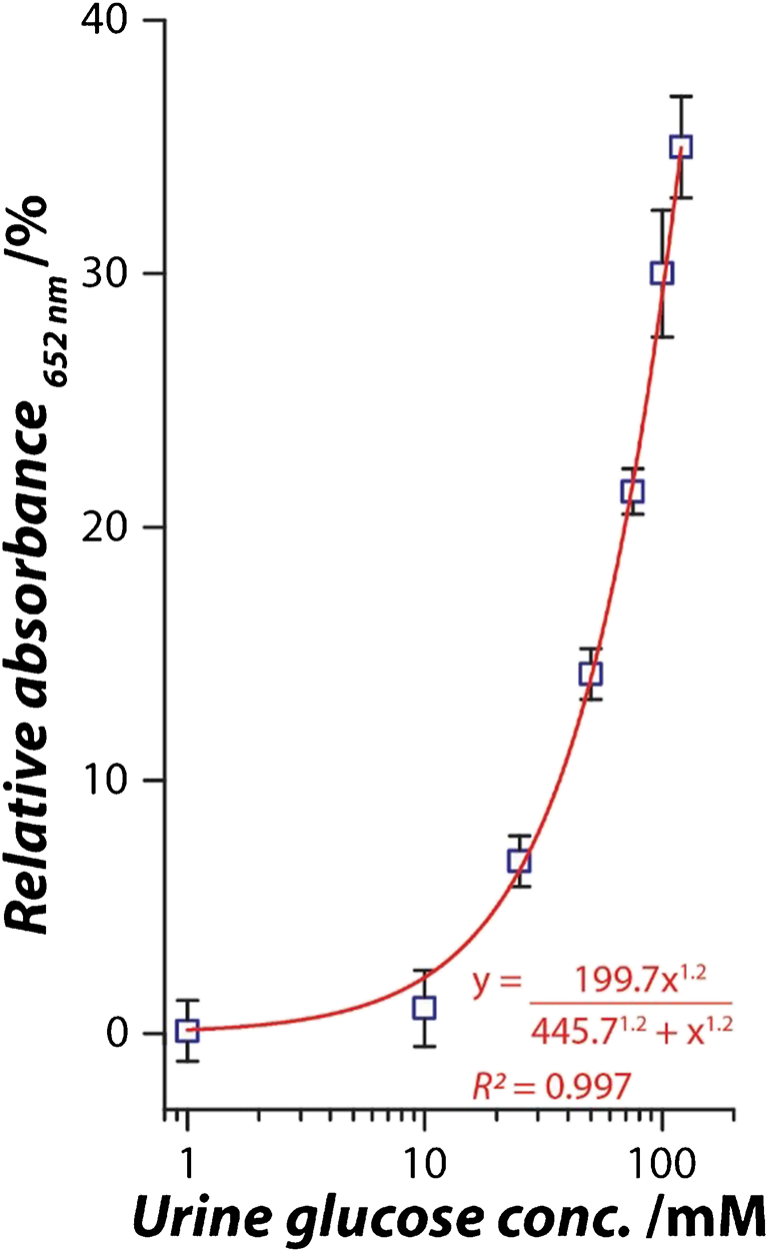
Urinary glucose sensing capabilities of Ag-Pt nanozyme fabric, presented as a % increase in the colorimetric signal upon exposure of the sensor to undiluted human urine spiked with different concentrations of glucose in the physiologically relevant range

Having established the linear dynamic range of the Ag-Pt nanozyme fabric sensor, its practical applicability in detecting glucose in human urine samples in the physiologically relevant range was validated by first evaluating it against the laboratory gold standard approach of glucose oxidase and horseradish peroxidase (GOx-HRP). For this, urine samples were collected from healthy volunteers, and glucose concentration was quantified using the laboratory gold standard GOx-HRP method. This assay resulted in no colour generation, suggesting that the urine samples contained undetectable glucose concentrations. Therefore, these samples were considered to contain 0 mM of glucose. The urine sample from a healthy volunteer was spiked with glucose to achieve a range of 1–120 mM urine glucose concentration. Importantly, the urine was spiked by directly dissolving glucose powder to avoid urine dilution, as our focus was to resemble the aspects of the urinary glucose monitoring scenario faced by patients. If this urine predilution step could be avoided, this could make the assay more practically viable. Notably, during the assay, the urine and the glucose therein get spontaneously diluted as the spiked urine sample only forms 10% of the volume of the assay, while the remaining 90% is made up of GOx, TMB, and buffer. Therefore, during the assay the glucose concentration is 0.1–12 mM. The sensor showed a glucose concentration-dependent trend in the 0.1–12 mM range, similar to that observed for glucose detection in buffer (Fig. 3a). This operational range translates to 1–120 mM of urinary glucose (Fig. 4), which is well within the physiologically relevant urinary glucose concentration range, triggering a response only under diseased conditions (glucose concentration > 0.8 mM) [41, 42]. The comparison of Ag-Pt nanozyme fabric with the gold standard assay (GOx-HRP) that required an additional 10X urine dilution revealed comparable performance. This is reflected by the corresponding recoveries of 92–108% (GOx-HRP assay) and 96–104% (Ag-Pt nanozyme assay) (Table S4). The possibility of avoiding urine predilution in our assay reflects the translational capabilities of the Ag-Pt nanozyme fabric as a portable point-of-care sensing platform.

Additionally, the practical applicability of the Ag-Pt nanozyme sensor was assessed by quantifying the concentration of glucose in the urine samples of healthy and diabetic volunteers (type II diabetes). The % recovery of the nanozyme sensor was calculated with reference to the glucose concentration estimated using the laboratory gold standard assay (GOx-HRP). The Ag-Pt fabric platform could reliably estimate the glucose concentration in both healthy and diabetic volunteer urine samples with a recovery of 97–105% (Table 1).

**Table 1:** Urinary glucose estimation in volunteer urine samples using the gold standard enzyme-only assay and the Ag-Pt nanozyme fabric assay

Volunteer urine samples	GOx-HRP approach^a^	Ag-Pt fabric approach^b^	
	Estimated urine glucose conc./mM^c,e^	Estimated urine glucose conc./mM^d,e^	Recovery/%^f^
Healthy—1	0.0 ± 0.00^g^	0.0 ± 0.00	100
Healthy—2	0.0 ± 0.00^g^	0.0 ± 0.00	100
Diabetic—1	3.13 ± 0.1	3.2 ± 0.09	102–103
Diabetic—2	1.2 ± 0.03	1.21 ± 0.08	97–105
Diabetic—3	11.29 ± 0.4	11.25 ± 0.2	98–101

^a^Urine was prediluted 10X prior to the GOx-HRP assay. The urine further underwent 10X dilution, as the urinary sample volumes were 10% of the total assay volume. Therefore, the effective urine dilution during the GOx-HRP assay was 100X

^b^Urine was not prediluted prior to the Ag-Pt nanozyme fabric assay. The urine underwent 10X dilution, as the urinary sample volumes were 10% of the total assay volume. Therefore, the effective urine dilution during the Ag-Pt nanozyme fabric assay was 10X

^c^Urine glucose concentration was determined after considering the dilution factor of 100X

^d^Urine glucose concentration was determined after considering the dilution factor of 10X

^e^Standard deviation calculated from three independent experiments

^f^Recovery calculated as (measured concentration/expected concentration) × 100

^g^Glucose undetectable

## Conclusions

This study demonstrates the ability to incorporate bimetallic Ag-M nanoparticles (M = Au, Pd, or Pt) into cotton fabrics via a combination of electroless deposition and galvanic replacement reactions. The addition of a second metal to the parent Ag fabrics expands the diversity in the surface composition, leading to the enhancement of the catalytic performance of the bimetallic fabrics. In the context of the relative activities of the bimetallic fabrics, the Ag-Pt nanozyme fabric showed the highest peroxidase-mimicking catalytic activity, accompanied by the lowest oxidase-mimicking activity. While high peroxidase activity is critical to achieving a stronger colorimetric response, low oxidase activity is desirable to avoid cross-reactivity of the sensor with other non-target species in biological matrices. Mechanistic insights into the Ag-Pt nanozyme revealed its ability to generate multiple ROS, including •OH and O_2_^•−^ radicals, which could be attributed to a free radical cascade supported by the transient oxidation of Ag atoms in this bimetallic system. The presence of Pt at the Ag-Pt interface may facilitate this process, leading to a synergistic effect. As the Ag-Pt nanozyme fabric could generate stronger colour response rapidly, it was converted into a colorimetric urinary glucose sensor by combining it with glucose oxidase. The Ag-Pt nanozyme fabric sensor could directly detect glucose in human urine in the 1–120 mM concentration range without requiring urine predilution. This dynamic operational range covers pathophysiological glucose levels in the urine of both humans and pets, where the latter also faces substantial challenges with diabetic monitoring. Notably, the interference effects from complex urine matrices are one of the key technological challenges in developing urinary sensors. Therefore, the ability to detect urinary glucose in the physiologically relevant range without requiring urine dilution attests to the robustness of the proposed sensor and outlines its potential from a practical perspective. These aspects point to the translational potential of the Ag-Pt nanozyme fabric as a point-of-care monitoring and detection technology.

## Supplementary Information

Below is the link to the electronic supplementary material.

Supplementary file1 (DOCX 2092 KB)

## Data Availability

Additional data are available in the Electronic Supplementary Material.

## Declarations

### Source of biological material

Urine samples from healthy and diabetic individuals were collected at RMIT University. All volunteers were provided with a detailed description of the study, and signed informed consent was obtained from each volunteer before urine samples were collected. The volunteers were requested to disclose their current health status—if they have been diagnosed as diabetic.
