# Supplementary material for: Silver-based bimetallic nanozyme fabrics with peroxidase-mimic activity for urinary glucose detection
Source: Anal Bioanal Chem. 2024 Aug 17;416(27):6149–59. doi: 10.1007/s00216-024-05483-7 (PMC11511708; doi:10.1007/s00216-024-05483-7)
Supplement: Supplementary file 1 — Supplementary file1 (DOCX 2092 KB) [file 216_2024_5483_MOESM1_ESM.docx]

**Electronic Supplementary Material**

**Silver-based bimetallic nanozyme fabrics with peroxidase-mimic activity for urinary glucose detection**

Sanjana Naveen Prasad,^a^ Sanje Mahasivam,^a^ Rajesh Ramanathan^a,^* and Vipul Bansal^a,^*

^a^Sir Ian Potter NanoBioSensing Facility, NanoBiotechnology Research Laboratory (NBRL), School of Science, RMIT University, Melbourne VIC 3000, Australia.

*E-mail: rajesh.ramanathan@rmit.edu.au; Phone: +61 3 9925 2887

*E-mail: vipul.bansal@rmit.edu.au; Phone: +61 3 9925 2121

# S1 Materials

Tin(II) chloride (SnCl_2_), palladium(II) nitrate (Pd(NO_3_)_2_), silver nitrate (AgNO_3_), ammonia (2.8% w/w aq.), gold(III) chloride hydrate (HAuCl_4_), chloroplatinic acid (8 wt.% in H_2_O) (H_2_Cl_6_Pt), palladium(II) chloride (PdCl_2_), acetic acid, anhydrous sodium acetate, 3,3’5,5’-tetramethylbenzidine (TMB), o-phenylenediamine dihydrochloride (OPD), 2,2′-azino-bis(3-ethylbenzothiazoline-6-sulphonic acid) (ABTS), terephthalic acid (TA), dihydroethidium (HE), 9,10-Anthracenediyl-bis(methylene)dimalonic acid (ABDA), glucose oxidase from *Aspergillus niger* (GOx from *Aspergillus niger*), horseradish peroxidase (HRP), glucose, fructose, galactose, lactose, sucrose, and maltose were purchased from Merck Australia. Milli-Q water (18.2 MΩ cm) was obtained from a Millipore Milli-Q water purification system. Cotton fabric was purchased from a local market, and hydrogen peroxide (30% w/w) was purchased from Chem-Supply Pty Ltd.

# S2 Synthesis of bimetallic Ag-M nanozyme fabrics (M = Au, Pd, Pt)

## S2.1 Methodology

Twenty pieces of 1 cm × 1 cm cotton fabric were sensitised in a 3 mM solution of SnCl_2_ for one hour, followed by exposure of the fabric to a 3 mM solution of Pd(NO_3_)_2_ for 30 min to form an initial seed layer of Pd^0^ nuclei. This was followed by washing the fabrics with Milli-Q water and further exposing them to a silver-plating solution containing equimolar quantities of glucose and diamine silver(I) complex ([Ag(NH_3_)_2_]^+^) for 15 min at room temperature. The resultant material contained Ag nanoparticles deposited within the 3D matrix of the cotton fabric, which was then washed with Milli-Q water to remove any free metal ions. These Ag fabrics were further used in the galvanic replacement (GR) reactions to produce bimetallic nanozyme fabrics.

One piece of the 1 cm × 1 cm Ag fabric was immersed in a 1 mM solution of HAuCl_4_, PdCl_2_, or H_2_PtCl_6_ for 1 h at room temperature. As a result of the spontaneous GR reaction, the resultant material (Ag-M fabric) contained Ag-M bimetallic nanoparticles (where M represents Au, Pd, or Pt) deposited onto original Ag particles embedded within the 3D matrix of cotton. The fabrics were washed several times with Milli-Q water to remove free ions, dried, and stored in a desiccator. The concentration of metal ions deposited on the surface of the fabric was quantified by atomic emission spectroscopy (AES) using an Agilent Technologies 4200 Microwave Plasma AES instrument.


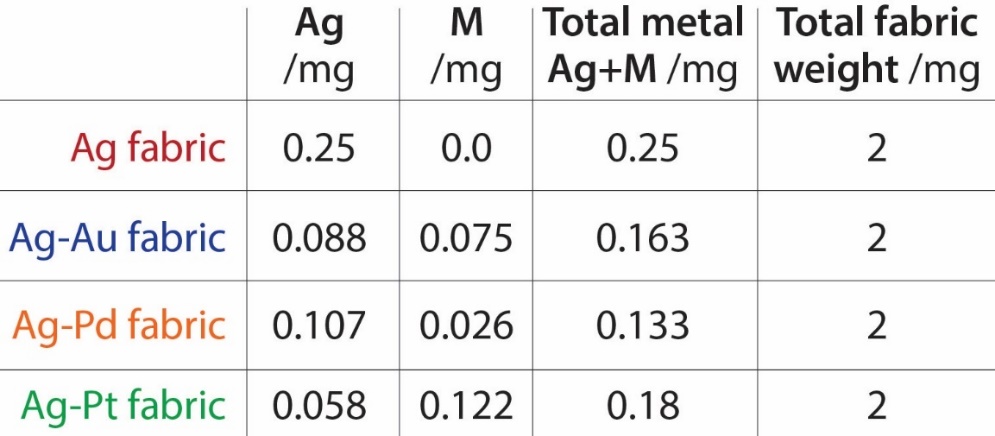


***Fig. S1.*** *Weights of Ag and noble metals present in 2 mg Ag-M fabrics.*

# S3 Characterisation of Ag-M nanozyme fabrics

## S3.1 Methodology

Morphological analysis of the Ag-M nanostructures was performed using an FEI Verios scanning electron microscope (operated at an accelerating voltage of 30 kV). Energy dispersive X-ray (EDX) spectroscopic analysis was performed on the same instrument using the Oxford X-Max 20 Silicon Drift detector. X-ray photoelectron spectroscopy (XPS) measurements were performed using a Thermo K-Alpha XPS instrument (Al Kα radiation, photon energy of 1486.6 eV). The C 1*s*, O 1*s*, Au 4*f*, Ag 3*d*, Pd 3*d*, and Pt 4*f* core level spectra were collected, and background corrected using the Shirley algorithm. Chemically distinct species were resolved using a nonlinear least square fitting procedure and were aligned with the adventitious C 1*s* binding energy of 285 eV.

## S3.2 Results and discussion

Scanning electron microscopy (SEM) images of the Ag-M fabrics clearly show the deposition of nanoparticles on the individual threads of the cotton fabrics (**Fig. S3** a). The parent Ag fabric showed quasi-spherical Ag nanoparticles of sub-100 nm in size, as shown in our previous study (**Fig. S2**) [1]. SEM images obtained after the GR reactions revealed an increase in surface roughness due to the deposition of Au, Pd, or Pt metal. Although the overall particle size remained within 100 nm, clusters of nanoparticles were present on the surfaces of the individual threads of the cotton fabric (**Fig. S3** a inset).


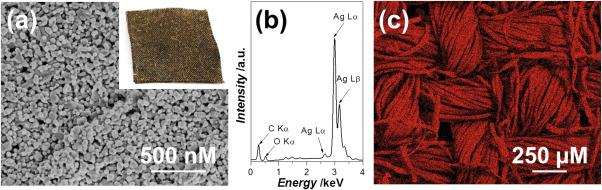


***Fig. S2.*** *SEM image along with a photograph (inset) of an Ag fabric (Reprinted with permission from [1], Copyright 2018, Elsevier).*

Energy-dispersive X-ray (EDX) spectral analysis of these hybrid fabrics showed the presence of characteristic energy lines associated with Ag Lα (2.98 keV and 3.15 keV), as well as those from the additional metal in the bimetallic nanostructures (**Fig. S3** b). For example, the Ag-Au fabric showed Au Mα energy line at 2.123 keV (**Fig. S3** b1) and the Ag-Pd fabric showed Pd Lα lines at 2.66 keV, 2.99 keV, and 3.55 keV (**Fig. S3** b2). The EDX elemental maps also indicated the uniform distribution of the bimetallic nanostructures on the surface of these hybrid fabric (**Fig. S3** c).

The oxidation states of the metals on the Ag-M fabrics were analysed using X-ray photoemission spectroscopy (XPS). All core level spectra were background corrected and their binding energies (BEs) were aligned to the adventitious C 1*s* BE of 285 eV. The core level Ag 3*d* spectrum from all Ag-M fabrics revealed two characteristic core level splitting components, 3*d_5/2_* and 3*d_3/2_* (spin-orbit splitting of ~6 eV) with a 3*d_5/2_* binding energy of 368.5±0.1 eV [2], corresponding to Ag in the zerovalent oxidation state (**Fig. S3** d). A minor peak observed at low binding energy is due to the plasmon loss feature of the Ag nanoparticles [3]. Additionally, Au 4*f* and Pd 3*d* core levels were detected, depending on the metal ions used during the GR reaction (**Fig. S3** e). The Ag-Au fabric exhibited characteristic 4*f_5/2_* and 4*f_3/2_* splitting components (spin-orbit splitting ~6 eV) with a 4*f_5/2_* component at *ca.* 84.2 eV which corresponds to Au^0^ (**Fig. S3** e1). No evidence of ionic gold was observed in this case. The Ag-Pd fabric showed two characteristic 3*d_5/2_* and 3*d_3/2_* splitting components (spin-orbit splitting ~5.3 eV). Spectral deconvolution and peak fitting revealed that in addition to the predominant Pd 3*d_5/2_* core level at ~335.6 eV, which corresponds to metallic Pd^0^, a minor component at ~338.2 eV due to Pd^2+^ is also present [4] (**Fig. S3** e2). This small amount of Pd^2+^ may be attributed to residual salt on the fabric surface from the GR reaction. Overall, the XPS analysis revealed that the Ag-M fabrics predominantly contained metallic forms of Ag and M, with minor ionic impurities in the case of Pd and Pt.


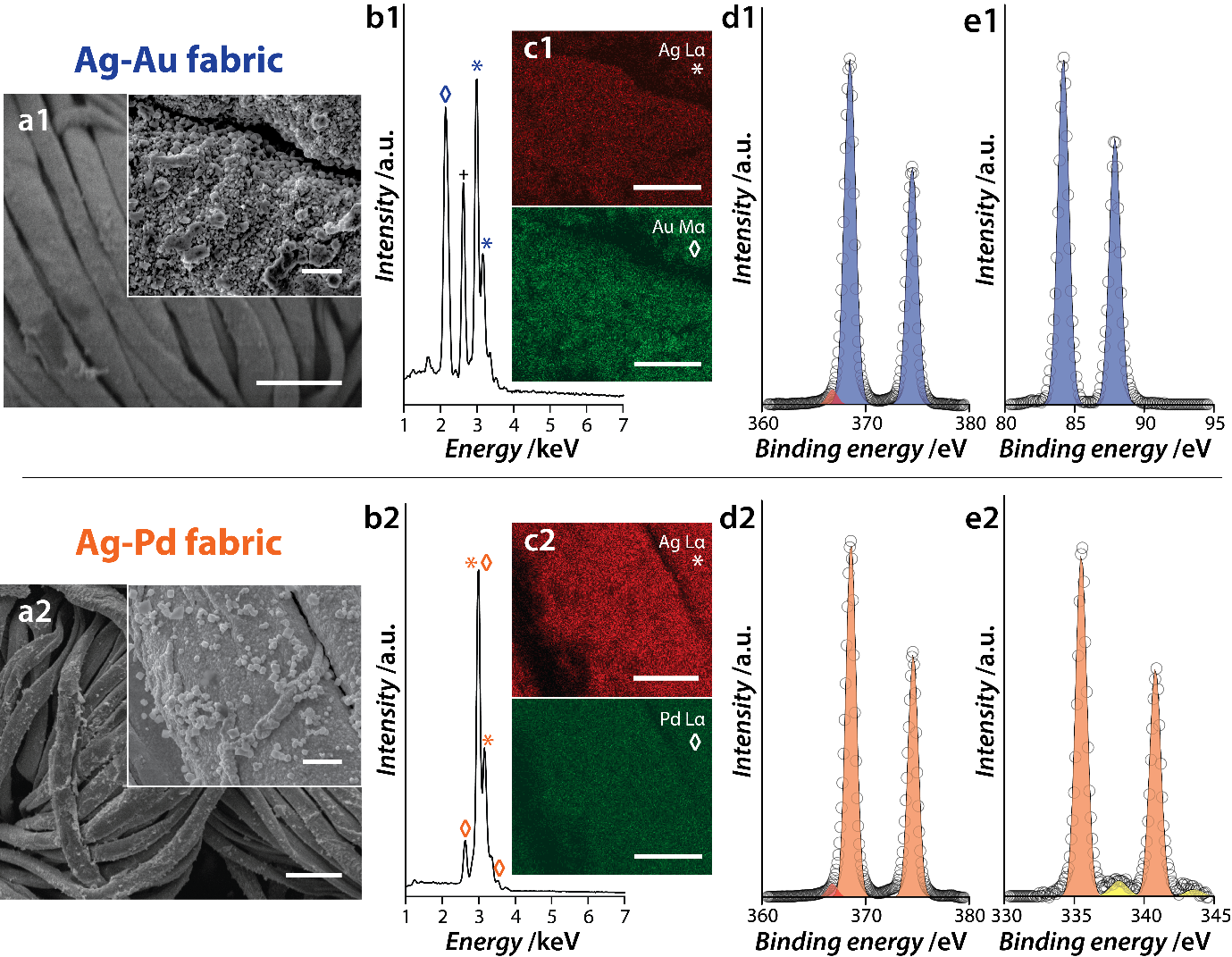


**Fig. S3.** Characterisation of the Ag-M fabrics, including **(a)** SEM images (scale bars correspond to 50 μm for the main figures and 5 μm for the insets); **(b)** EDX spectra where * represents Ag, ◊ represents the GR metal, and + represents residual Cl; **(c)** EDX maps showing the distribution of the different metals (scale bars correspond to 10 μm); **(d)** Ag 3d XPS core level spectra and **(e)** Au 4f and Pd 3d XPS core level spectra obtained from the respective Ag-M bimetallic fabrics.

# S4 Peroxidase- and oxidase- mimic catalytic activities of Ag and Ag-M nanozyme fabrics

## S4.1 Methodology

The peroxidase and oxidase-mimicking catalytic activities of the Ag-M nanozyme fabrics and the parent Ag nanozyme fabric were studied by assessing their capability to oxidise the chromogenic TMB substrate in the presence and absence of H_2_O_2_ as a co-substrate, respectively. The assays were carried out at 37 °C in 50 mM sodium acetate buffer at pH 5 with 0.2 mM TMB, 10 mM H_2_O_2_ and 2 mg of the nanozyme fabric. For the oxidase-mimicking assay, H_2_O_2_ was excluded.


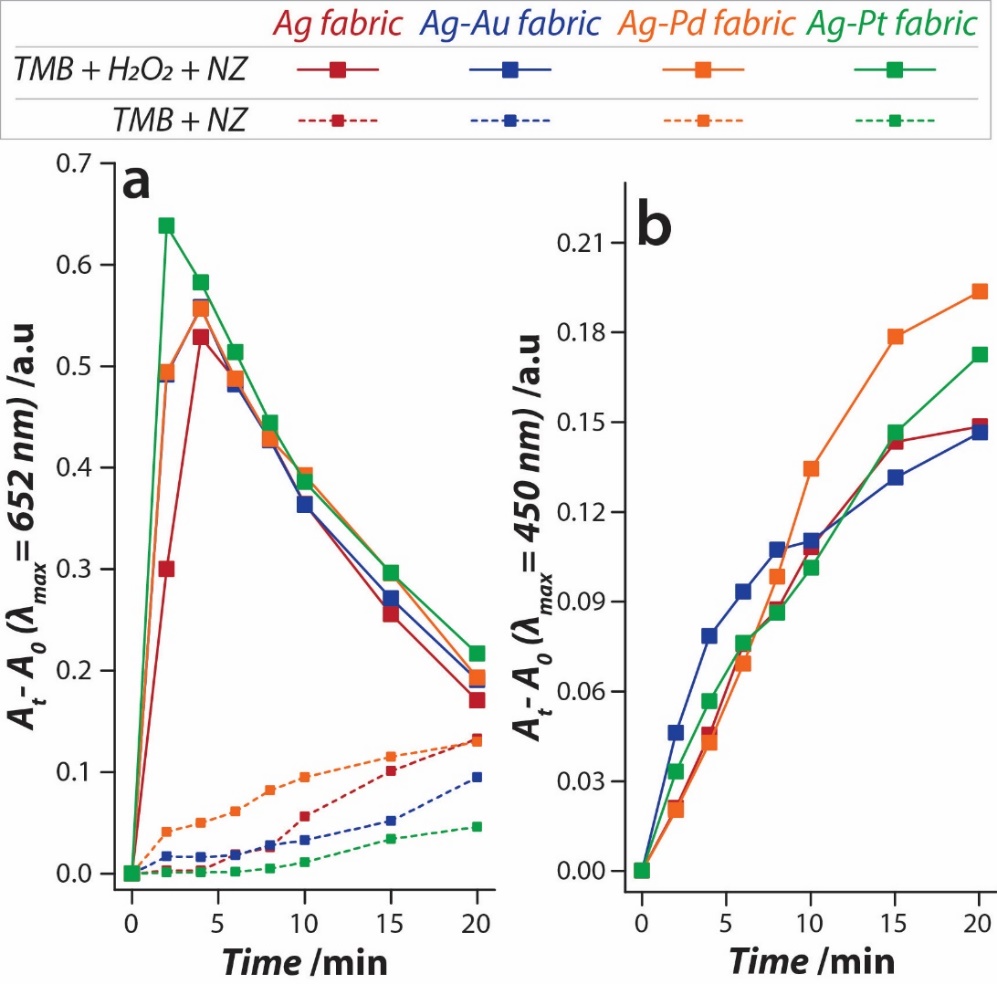


***Fig. S4.*** *Comparison of the time-dependent nanozyme activity of the Ag and Ag-M fabrics (2 mg).* ***(a)*** *shows the generation of the blue product of TMB oxidation as a result of peroxidase (TMB+H_2_O_2_+NZ, solid line ■) and oxidase (TMB+ NZ, dotted line ■) mimicking catalytic activities.* ***(b)*** *shows the generation of yellow double-oxidation product of TMB directly without adding an acid to the reaction due to the outstanding peroxidase-mimicking activity of these nanozyme fabrics. (Reaction conditions: pH – 5, temperature – 37 °C, concentration of TMB – 0.2 mM, concentration of H_2_O_2_ – 10 mM).*

To determine the rates of reaction, the assays were carried out in 50 mM sodium acetate buffer (pH 5) with 0.2 mM TMB, 10 mM H_2_O_2_ and 1 mg of the nanozyme fabric, incubated at 37 °C. Time-dependent UV-vis absorbance spectroscopy measurements of the assays were carried out on a CLARIOstar plate reader (BMG Labtech). Absorbance measurements were plotted as *ln*(A_t_/A_0_) *vs*. time (where A_t_ is the absorbance at time t and A_0_ is the absorbance at 0 min) to derive the reaction rates from the linear regions of the curves, which was then normalised to the active weight of the metal on a 1 mg piece of Ag or Ag-M nanozyme fabric.


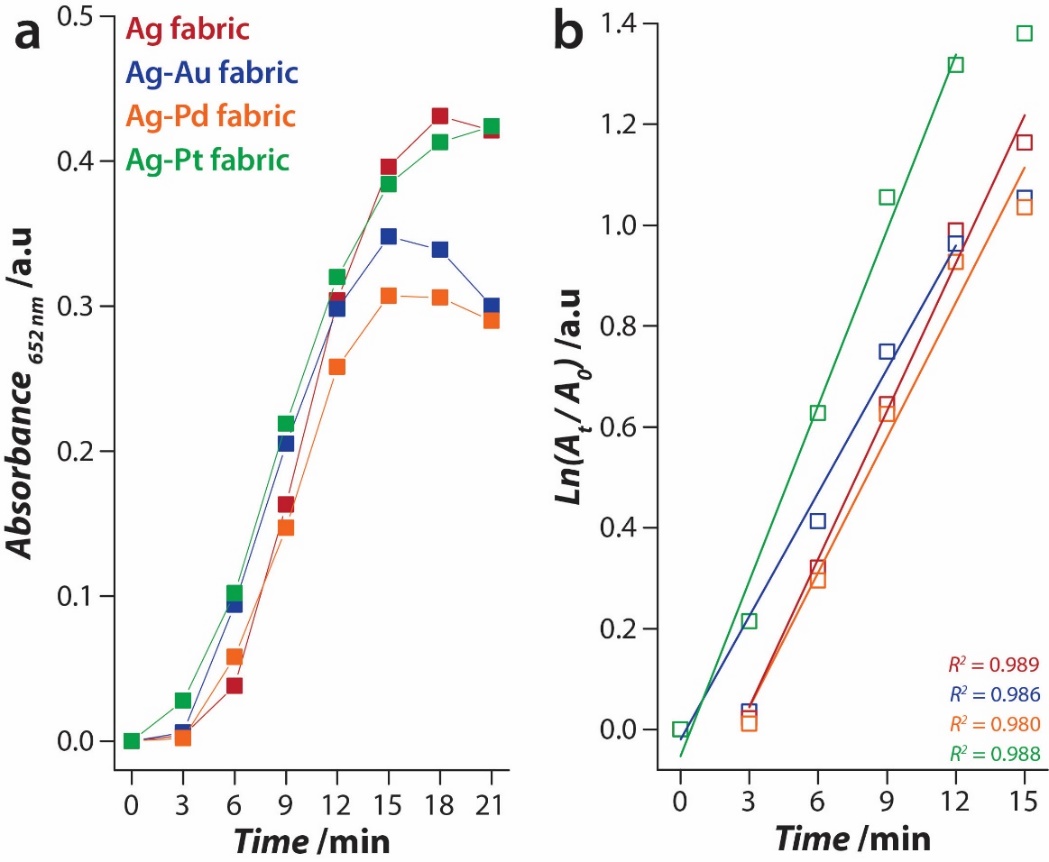


***Fig. S5****.* ***(a)*** *TMB oxidation product formation by Ag and Ag-M fabrics weighing 1 mg (Reaction conditions: pH – 5, temperature – 37 °C, concentration of TMB – 0.2 mM, concentration of H_2_O_2_ – 10 mM).* ***(b)*** *Plot of ln(A_t_/A_0_) as a function of time to derive the rates of TMB oxidation from the linear portion of the curve.*

# S5 Standardization of nanozyme assay parameters for Ag-Pt fabric

## S5.1 Methodology

Among the three Ag-M fabrics, the Ag-Pt nanozyme was chosen for further study due to the high catalytic activity of this system. Ag-Pt nanozyme fabric eventually led to the development of a urinary glucose sensor. Various assay parameters, such as the choice of substrate, nanozyme concentration, reaction pH, and temperature, were standardised. First, the substrate specificity of the Ag-Pt nanozyme fabric was assessed by exposing the fabric to the chromogenic substrates TMB, OPD, and ABTS, followed by spectroscopic quantification of their respective oxidation products using Beer-Lambert’s law (extinction coefficients: TMB_652 nm_ = 39000 M^-1^cm^-1^, OPD_417 nm_ = 16700 M^-1^cm^-1^, ABTS_420 nm_ = 36000 M^-1^cm^-1^ [5]). The potential nanozyme activity of leached metal ions in solution was assessed by incubating the Ag-Pt fabric in pH 5 buffer for 20 min at 37°C, followed by extraction of the Ag-Pt fabric while adding 0.2 mM TMB and 10 mM H_2_O_2_ to the buffer solution containing potential leached ions. The optimum reaction pH and temperature were determined by independently varying the reaction pH (1.0-12.0) and temperature (25-60 °C).

## S5.2 Results and discussion

The intrinsic peroxidase-mimicking catalytic activity of the Ag-Pt fabric was validated by ruling out the possibility of free metal ions potentially leaching out of these fabrics, catalysing the oxidation of TMB. To ascertain this, the Ag-Pt fabric was exposed to a buffer solution for 20 min. Subsequently, the fabric was extracted and the buffer containing any possible leached metal ions (Ag and Pt) was used as a catalyst. No TMB oxidation product was observed, confirming the intrinsic catalytic activity of the Ag-Pt nanozyme (**Fig. S6** a). Optimisation of the assay parameters was then carried out to facilitate the development of a glucose sensing platform based on the Ag-Pt nanozyme. Typically, the catalytic efficiency of a nanozyme depends on the substrate used during the reaction, reaction temperature, and reaction pH. Hence, these parameters were optimised.

To assess the influence of the substrate on the reaction, the catalytic activity of the Ag-Pt nanozyme was assessed against other commonly used peroxidase substrates, such as o-phenylenediamine dihydrochloride (OPD; λ_max_ = 417 nm) and 2,2′-azino-bis(3-ethylbenzothiazoline-6-sulphonic acid) (ABTS; λ_max_ = 420 nm). Compared to TMB, the concentration of the oxidised products of the other substrates was ~90% lower (**Fig. S6** b), making TMB the preferred substrate.


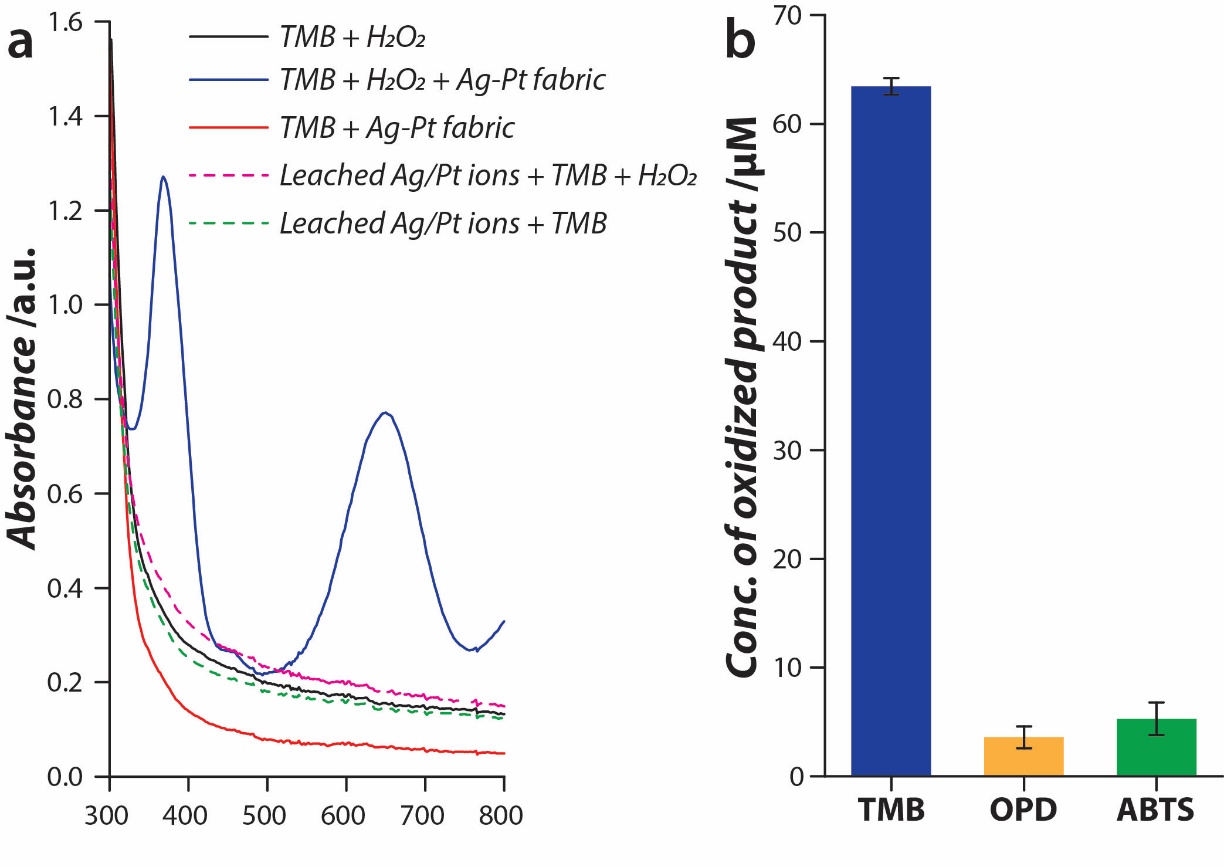


***Fig. S6.*** ***(a)*** *Absorbance spectra of Ag-Pt catalysed TMB oxidation reaction under different conditions to assess the role of potentially leached metal ions in catalysis;* ***(b)*** *Relative concentrations of products obtained from the oxidation of different colorimetric substrates of peroxidase-mimic Ag-Pt nanozyme fabric. (Reaction conditions: pH – 5, temperature – 37 °C, concentration of colorimetric substrate – 0.2 mM, concentration of H_2_O_2_ – 10 mM, weight of Ag-Pt fabric – 2 mg, reaction time – 2 min). The concentrations of oxidized products were determined using Beer-Lambert’s law.*

Next, the pH dependence of the Ag-Pt nanozyme was evaluated, which revealed optimal activity at slightly acidic pH values of 4 and 5, with a 50-70% reduction in activity at pH 3 and 6, respectively (**Fig. S7** a). This is consistent with other peroxidase-mimicking nanozymes that typically show high catalytic activity for TMB oxidation under slightly acidic conditions [1, 6, 7]. Inorganic catalysts typically exhibit higher catalytic activities as the reaction temperature increases. However, the Ag-Pt fabric revealed an enzyme-like behaviour, as the catalytic activity was reduced at low and high temperatures (**Fig. S7** b). The optimal catalytic activity was observed at 37 °C. Therefore, the optimum assay parameters for the Ag-Pt nanozyme fabric were determined to be 37 °C and pH 5 while using TMB as the substrate.


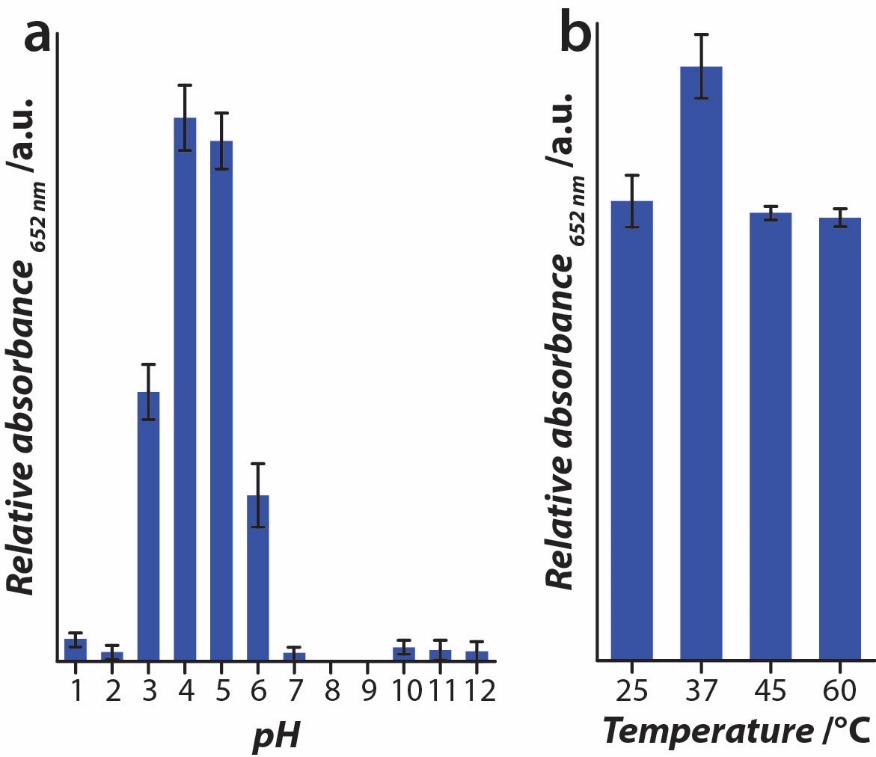


***Fig. S7.*** *Effect of* ***(a)*** *pH and* ***(b)*** *temperature on the peroxidase-mimic catalytic activity of Ag-Pt nanozyme fabric. The reactions were carried out at pH 5 for* ***(b)*** *and at a temperature of 37 °C for* ***(a)****. (Reaction conditions: concentration of TMB – 0.2 mM, concentration of H_2_O_2_ – 10 mM, weight of Ag-Pt fabric – 2 mg, reaction time – 2 min).*

# S6 Steady-state kinetic parameters of Ag-Pt nanozyme fabric

## S6.1 Methodology

The kinetic parameters such as the Michaelis constant (*K_m_*) and maximum reaction velocity (V_max_) of an enzyme-mimicking reaction were determined for a fixed weight of the bimetallic Ag-Pt nanozyme fabric (2 mg), while independently varying the concentration of TMB and H_2_O_2_. The reactions were carried out at 37 °C in pH 5 buffer. The colorimetric response was then fitted to a Lineweaver-Burk plot using OriginPro2016, followed by calculation of the *K_m_* and *V_max_* values using Equation S1.

$\frac{1}{V_{0}}=\frac{K_{m}}{V_{max}}\left( \frac{1}{\left[ S \right]}+\frac{1}{K_{m}} \right)$ (Equation S1)

where *V_0_* corresponds to the initial reaction velocity, *K_m_* is the Michaelis-Menten constant, *V_max_* is the maximum reaction velocity, and [S] is the substrate concentration.

## S6.2 Results and discussion

For natural organic enzymes, the Michaelis constant (*K_m_*) and maximum initial velocity of reaction (*V_max_*) are two steady-state kinetic parameters used to evaluate their catalytic activity. Here, *K_m_* is indicative of substrate affinity, whereas *V_max_* shows how quickly the reaction proceeds [5]. As the Ag-Pt nanozyme fabric showed catalytic behaviour similar to natural enzymes, the *K_m_* and *V_max_* were determined for this nanozyme. These parameters also provide insights into the suitability of the Ag-Pt nanozyme as a potential candidate for developing a sensor for glucose detection. The peroxidase-mimic catalytic reactions involve TMB and H_2_O_2_ as co-substrates, and therefore to understand the overall reaction mechanism, the kinetic parameters were determined for both substrates using Lineweaver-Burk plots (**Fig. S8**).


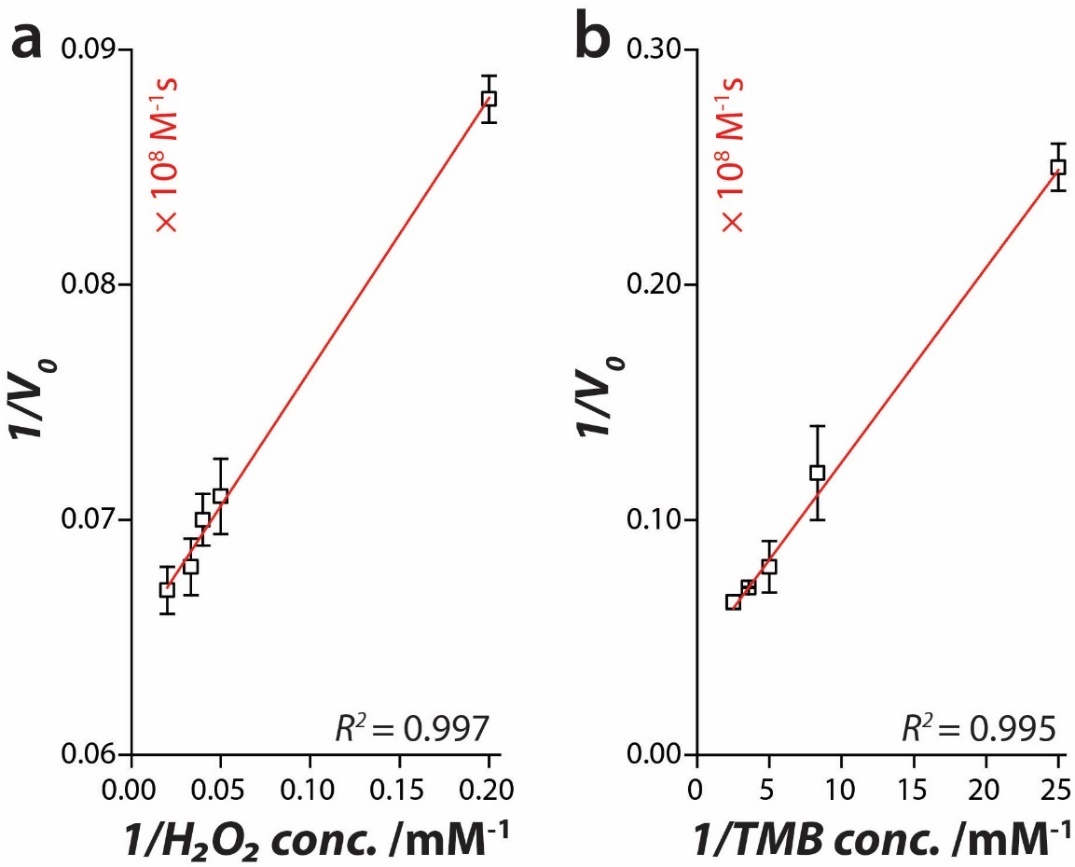


***Fig. S8****. Steady-state kinetic analysis using the Lineweaver-Burk fit for the colorimetric response obtained by varying the concentrations of* ***(a)*** *H_2_O_2_ and* ***(b)*** *TMB while keeping the concentration of the other substrate constant, that is, TMB (0.2 mM) and H_2_O_2_ (10 mM), respectively. (Reaction conditions: pH – 5, temperature – 37 °C, active metal weight in the Ag-Pt fabric – 0.18 mg, reaction time – 2 min).*

The apparent *K_m_* of the Ag-Pt nanozyme for TMB was found to be similar to that of the parent monometallic Ag nanozyme (**Table S1**). However, *V_max_* was ~1.6 times higher, implying that even with a similar affinity to TMB, bimetallic Ag-Pt nanozyme fabrics can promote a significantly higher rate of TMB oxidation. The ability of the Ag-Pt nanozyme to oxidise TMB more rapidly than the Ag nanozyme can be understood by comparing the *K_m_* of these two nanozymes towards H_2_O_2_. In a peroxidase-mimic reaction, the production of free radicals (typically •OH radicals) *via* catalyst-mediated decomposition of H_2_O_2_ is the first step in the TMB oxidation process. The *K_m_* of the Ag-Pt nanozyme for H_2_O_2_ is significantly lower (less than one-fourth of that of the Ag nanozyme), indicating a higher affinity of the Ag-Pt nanozyme to H_2_O_2_. This high affinity can overcome kinetic diffusion barriers during the reaction, making H_2_O_2_ more accessible to the Ag-Pt nanozyme fabric during the reaction. Even with a nearly similar *V_max_* of both these catalysts towards H_2_O_2_, the ability of the Ag-Pt nanozyme to overcome diffusion barriers can facilitate rapid access of free radicals to promote faster TMB oxidation. Further, the significantly low *K_m_* of the Ag-Pt nanozyme for H_2_O_2_ also means that even at a lower concentration of H_2_O_2_, the Ag-Pt nanozyme can generate a strong colour response *via* TMB oxidation. We note that the production of intense colours is a key performance characteristic of high-quality colorimetric sensor technologies, and if the use of H_2_O_2_ can be minimised in these sensors, it offers an invaluable advantage, as high concentrations of H_2_O_2_ typically contribute to undesirable issues related to sensor robustness and specificity.

***Table S1.*** *Comparison of the apparent Michaelis-Menten constant (K_m_) and maximum rate of reaction (V_max_) for Cu and Ag-based nanozyme fabrics.*

| **Catalyst** | **TMB** | | **H_2_O_2_** | |
| --- | --- | --- | --- | --- |
|  | ***K_m_* /mM** | ***V_max_* /×10^-8^ Ms^-1^** | ***K_m_* /mM** | ***V_max_* /×10^-8^ Ms^-1^** |
| Cu nanozyme [7] | 0.25 | 21.5 | 7.9 | 10.3 |
| Cu-Pt nanozyme [6] | 0.27 | 13.6 | 0.9 | 7.1 |
| Ag nanozyme [1] | 0.19 | 15.1 | 7.61 | 14.4 |
| Ag-Pt nanozyme  (current work) | 0.19 | 24.0 | 1.78 | 15.43 |

We also compared the steady-state kinetic parameters of the Ag and Ag-Pt fabrics with those of Cu and Cu-Pt fabrics previously developed by our team for urinalysis [6]. The comparison reveals that upon conversion of monometallic fabrics into bimetallic fabrics, similar trends of changes are seen for the *K_m_* for both Ag and Cu systems. However, the Ag-based fabrics outperform the Cu-based fabrics in driving the rate of reaction. While the conversion of Ag to Ag-Pt leads to an increase in the *V_max_* of the reaction for both TMB and H_2_O_2_ oxidation, in the case of the Cu fabric, a reduction in the *V_max_* is observed when these were converted into Cu-Pt fabrics. These aspects clearly outline the superiority of the Ag-Pt nanozyme fabrics in driving peroxidase-mimic reactions.

# S7 Mechanism of peroxidase-mimicking catalytic activity of Ag-Pt nanozyme fabric

## S7.1 Methodology

The underlying mechanism by which the Ag-Pt fabric exhibited peroxidase-mimicking catalytic activity was determined by investigating the generation of three reactive oxygen species (ROS) – hydroxyl radicals (•OH), superoxide radicals (O_2_^•-^), and singlet oxygen (^1^O_2_), by the nanozyme in the presence of H_2_O_2_. Probes specific to each ROS were used, such as TA for •OH, HE for O_2_^•-^, and ABDA for ^1^O_2_. The •OH radicals produced during the degradation of H_2_O_2_ by the Ag-Pt nanozyme are captured by TA, forming a fluorescent 2-hydroxyterephthalic acid (excitation: 320 nm, emission: 350-550 nm). The O_2_^•-^ radicals generated in the Ag-Pt catalysed reaction was assayed using fluorescent HE which reacts with O_2_^•-^ to form 2-hydroxyethidium (excitation: 490 nm, emission: 540-740 nm). A colorimetric substrate, ABDA (absorbance maxima at 360, 380, and 400 nm) was used to determine the production of ^1^O_2_ species. The reactions involved 1 mM TA / 2 µM HE / 0.1 mM ABDA, 10 mM H_2_O_2_, and 2 mg Ag-Pt fabric in 50 mM NaAc buffer (pH 5) incubated at 37 °C for 10 min followed by spectral measurement on a CLARIOstar plate reader (BMG Labtech).

## S7.2 Results and discussion

The typical mechanism of the catalytic decomposition of H_2_O_2_ and other peroxides by natural peroxidases such as HRP involves the production of •OH radicals [8]. Similarly, most peroxidase-mimicking nanozymes generate •OH radicals, which eventually oxidise the chromogenic and fluorometric substrates [1, 6, 7, 9-12]. However, in certain cases, natural peroxidases have been observed to facilitate alternative or additional atypical reactions through which other ROS, including O_2_^•-^ radicals and ^1^O_2_ species, may be produced [13, 14]. To determine which ROS are produced during the peroxidase-mimic catalytic activity of the Ag-Pt nanozyme fabric, probes specific to each ROS were used. The generation of •OH radicals was determined using a fluorogenic probe, terephthalic acid (TA), which oxidises to form a fluorescent product, 2-hydroxyterephthalic acid, upon binding to •OH radicals [6]. When the Ag-Pt fabric was exposed to H_2_O_2_, an emission peak was observed at 420 nm (**Fig. S9** a), suggesting that the Ag-Pt nanozyme was capable of catalysing the breakdown of the O–O bond in H_2_O_2_ to generate •OH radicals [15]. The potential of the Ag-Pt nanozyme in the generation of O_2_^•-^ radicals was assessed using the fluorogenic probe hydroethidine (HE), which upon oxidation by O_2_^•-^ radicals, is converted to fluorescent 2-hydroxyethidium with an emission maximum at 640 nm [16]. Interestingly, the Ag-Pt nanozyme could also generate O_2_^•-^ radicals when exposed to H_2_O_2_ (**Fig. S9** b). Subsequently, the potential formation of ^1^O_2_ species by Ag-Pt nanozyme was probed using a colorimetric probe, 9,10-anthracenediylbis(methylene)dimalonic acid (ABDA), with the expectation that the presence of ^1^O_2_ species should reduce the intensity of the key absorbance peaks of ABDA [17]. No significant change in the absorbance of ABDA suggests that the Ag-Pt nanozyme did not promote H_2_O_2_ decomposition *via* the formation of ^1^O_2_ species (**Fig. S9** c).


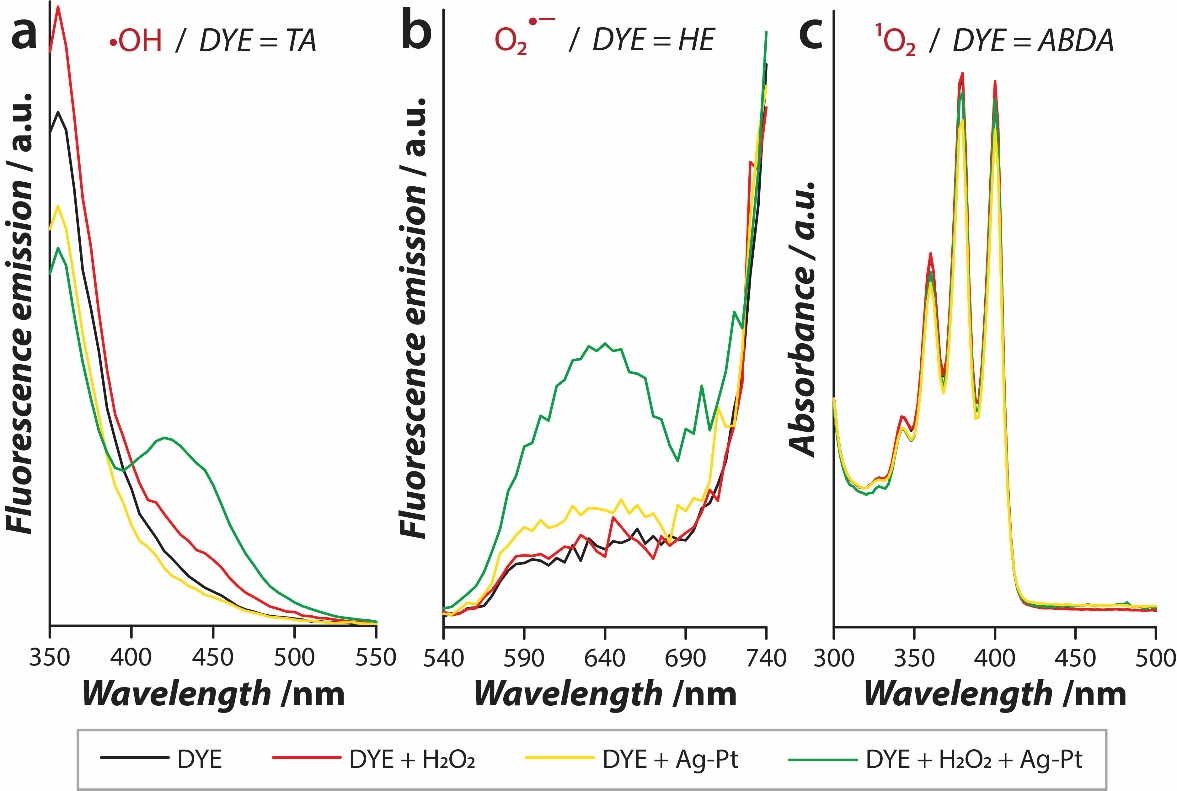


**Fig. S9**. Investigation of ROS generated by Ag-Pt nanozyme fabric. Fluorescence emission spectra of the **(a)** hydroxyl radical probe – TA and **(b)** superoxide radical probe – HE, and **(c)** UV-vis spectra of the singlet oxygen probe – ABDA, under different reaction conditions.

Overall, the Ag-Pt nanozyme fabric could facilitate the decomposition of H_2_O_2_ *via* the production of two ROS, •OH and O_2_^•-^ radicals. The simultaneous production of these ROS by the Ag-Pt nanozyme can be attributed to the classical Haber-Weiss mechanism [18, 19]. The mechanism involves a free radical chain reaction and has been proposed to account for the O_2_^•-^ radicals produced *via* the direct decomposition of H_2_O_2_ in certain transition metal catalysts [20]. It was noted that the efficiency of O_2_^•-^ radical production depended on the oxidation potential of the transition metal, such that the highest rate constant was observed for Ag, followed by Pt. Coincidentally, among the different bimetallic nanozymes investigated in our current study, the Ag-Pt system reveals the most profound peroxidase-mimic (H_2_O_2_ decomposition) activity. Therefore, we believe that the simultaneous production of •OH and O_2_^•-^ radicals by the Ag-Pt nanozyme fabric is achieved through a series of reactions presented in Equations (S2 – S5).

$AgPt+ H_{2}O_{2}\to{Ag}^{+}Pt+ {OH}^{-}+ \cdot OH$ (Equation S2)

$\cdot OH+ H_{2}O_{2}\to H_{2}O+ {\cdot HO}_{2}$ (Equation S3)

${\cdot HO}_{2}\rightleftharpoons H^{+}+ {O_{2}}^{.-}$ (Equation S4)

As the oxidation of Ag is easier than that of Pt, we believe that some of the Ag^0^ atoms are locally oxidized during the initial reduction of H_2_O_2_, and •OH radicals are produced (Equation S2). This reaction is akin to a typical peroxidase-mediated reaction that results in the formation of •OH radicals. Subsequently, the •OH radicals produced on the Ag-Pt surface can react with another H_2_O_2_ molecule to produce perhydroxyl radicals (Equation S3). These perhydroxyl radicals can dissociate into O_2_^•-^ radicals, but due to the reversibility of this reaction (Equation S4), an appropriate catalyst is critical to drive the reaction equilibrium in the forward direction. Since Ag and Pt offer the most appropriate oxidation potentials for this reaction [20], we observed the production of O_2_^•-^ radicals by the Ag-Pt nanozyme, which is typically not observed by other peroxidase-mimicking nanozymes. As noted above, certain plant peroxidases are also known to catalyse the generation of O_2_^•-^ radicals [14]. Although the underlying mechanism may not necessarily be the same as that proposed for the Ag-Pt nanozyme, most natural peroxidases are heme proteins and employ the redox Fe(III)/(II) couple for their activity. This suggests that the proposed nanozyme activity of the Ag-Pt system *via* a redox pathway (Equation S2) could offer some resemblance to natural peroxidases. Further, the O_2_^•-^ radicals produced from the decomposition of H_2_O_2_ (Equations S2-S4) can further react with the partially oxidized Ag^+^‑Pt to regenerate the original Ag-Pt nanozyme *via* Equation S5.

${O_{2}}^{.-}+ {Ag}^{+}Pt\to AgPt+ O_{2}$ (Equation S5)

This regeneration of the Ag-Pt nanozyme fulfils the key criteria for the definition of a catalyst/ enzyme/ enzyme-mimic, *i.e.*, the catalyst remains unused at the end of the reaction. The overall reaction derived from the series of steps outlined in Equations (S2 – S5) can be represented by Equation S6.

${2H}_{2}O_{2}\underset{\to}{AgPt} {OH}^{-}+ H_{2}O+ H^{+}+ O_{2}$ (Equation S6)

On balance, this shows that despite a series of intermediate steps involving a free radical chain reaction during the decomposition of H_2_O_2_, the composition of Ag-Pt does not change during the reaction, while at least some of these free radicals simultaneously participate in the oxidation of TMB molecules to convert them from colourless to blue.

# S8 Colorimetric detection of glucose using Ag-Pt nanozyme fabric

***Table S2.*** *Comparison of the glucose detection range of Cu and Ag-based nanozyme fabrics.*

| **Catalyst** | **Linear range** | **LoD** | **Accuracy**  (5% contingency) | **Precision** |
| --- | --- | --- | --- | --- |
| Cu nanozyme [7] | 0.5 – 15 mM | 0.45 mM | 93.3% | 96.8% |
| Cu-Pt nanozyme [6] | 1 – 12.5 mM | 0.84 mM | 92.8% | 97.1% |
| Ag nanozyme [1] | 0.1 – 2 mM | 0.08 mM | 100% | 94.7% |
| Ag-Pt nanozyme  (current work) | 0.1 – 12 mM | 0.06 mM | 93.3% | 96.5% |

***Table S3.*** *Batch-to-batch variation in glucose estimation using Ag-Pt nanozyme fabric.*

| **Batch** | **Glucose conc. /mM** | **Accuracy**^d^  (5% contingency) | **Precision**^e^ |
| --- | --- | --- | --- |
| In-batch^a^ | 0.1 | 100% | 97.3% |
|  | 1 | 100% | 97.7% |
|  | 12 | 100% | 97.3% |
| Intra-batch^b^ | 0.1 | 94.1% | 96.7% |
|  | 1 | 92.3% | 94.1% |
|  | 12 | 92.3% | 94.2% |
| Inter-batch^c^ | 0.1 | 91% | 94% |
|  | 1 | 91% | 95.3% |
|  | 12 | 92.8% | 94.9% |

*^a)^Glucose sensing assays conducted using multiple Ag-Pt fabrics synthesised simultaneously in a single batch.*

*^b)^ Glucose sensing assays conducted on different days using multiple Ag-Pt fabrics synthesised simultaneously in a single batch.*

*^c)^ Glucose sensing assays conducted using Ag-Pt fabrics synthesised in different independent batches.*

***Table S4.*** *Glucose estimation in urine samples obtained from healthy volunteers after spiking with predetermined concentrations of glucose. Comparison of results obtained using the gold standard enzyme-only assay (glucose oxidase + horseradish peroxidase) and the nanozyme approach (glucose oxidase + Ag-Pt fabric).*

| **Glucose spiked /mM** | **GOx-HRP approach**^a)^ | | | **Ag-Pt fabric approach**^b)^ | | |
| --- | --- | --- | --- | --- | --- | --- |
|  | **Expected glucose conc. /mM**^c)^ | **Estimated glucose conc. /mM**^e)^ | **Recovery /%**^f)^ | **Expected glucose conc. /mM**^d)^ | **Estimated. glucose conc. /mM**^e)^ | **Recovery /%**^f)^ |
| 0 | 0 | 0 ± 0.00 | 100 | 0 | 0 ± 0.00 | 100 |
| 1 | 0.01 | 0.01 ± 0.001 | 92-108 | 0.1 | 0.1 ± 0.004 | 96-104 |
| 10 | 0.1 | 0.1 ± 0.002 | 98-101 | 1 | 0.99 ± 0.02 | 97-101 |
| 50 | 0.5 | 0.51 ± 0.014 | 98-104 | 5 | 5 ± 0.01 | 100 |
| 100 | 1 | 1.01 ± 0.026 | 99-104 | 10 | 10.1 ± 0.15 | 100-103 |
| 120 | 1.2 | 1.21 ± 0.016 | 100-102 | 12 | 12.3 ± 0.18 | 101-104 |

*^a)^ Urine was pre-diluted 10X.*

*^b)^ Urine was not pre-diluted.*

*^c)^ The urine further underwent 10X dilution, as the urinary sample volumes were 10% of the total assay volume. Therefore, the effective urine dilution during the GOx-HRP assay was 100X.*

*^d)^ The urine underwent 10X dilution, as the urinary sample volumes were 10% of the total assay volume. Therefore, the effective urine dilution during the Ag-Pt nanozyme fabric assay was 10X.*

*^e)^ Standard deviation calculated from three independent experiments.*

*^f)^ Recovery calculated as (measured concentration/expected concentration) × 100.*

# S9 References

1. Karim MN, Anderson SR, Singh S, Ramanathan R, Bansal V. Nanostructured silver fabric as a free-standing NanoZyme for colorimetric detection of glucose in urine. Biosens Bioelectron. 2018;110:8-15. <https://doi.org/10.1016/j.bios.2018.03.025>

2. Gaarenstroom SW, Winograd N. Initial and final state effects in the ESCA spectra of cadmium and silver oxides. J Chem Phys. 2008;67(8):3500-6. <https://doi.org/10.1063/1.435347>

3. Leiro J, Minni E, Suoninen E. Study of plasmon structure in XPS spectra of silver and gold. J Phys F: Met Phys. 1983;13(1):215. <https://doi.org/10.1088/0305-4608/13/1/024>

4. Tressaud A, Khairoun S, Touhara H, Watanabe N. X-ray photoelectron spectroscopy of palladium fluorides. Z anorg allg Chem. 1986;540(9-10):291-9. <https://doi.org/10.1002/zaac.19865400932>

5. Jiang B, Duan D, Gao L, Zhou M, Fan K, Tang Y, et al. Standardized assays for determining the catalytic activity and kinetics of peroxidase-like nanozymes. Nat Protoc. 2018;13(7):1506. <https://doi.org/10.1038/s41596-018-0001-1>

6. Naveen Prasad S, Anderson SR, Joglekar MV, Hardikar AA, Bansal V, Ramanathan R. Bimetallic nanozyme mediated urine glucose monitoring through discriminant analysis of colorimetric signal. Biosens Bioelectron. 2022;212:114386. <https://doi.org/10.1016/j.bios.2022.114386>

7. Naveen Prasad S, Weerathunge P, Karim MN, Anderson S, Hashmi S, Mariathomas PD, et al. Non-invasive detection of glucose in human urine using a color-generating copper NanoZyme. Anal Bioanal Chem. 2021;413:1279–91. <https://doi.org/10.1007/s00216-020-03090-w>

8. Rodríguez-López JN, Lowe DJ, Hernández-Ruiz J, Hiner ANP, García-Cánovas F, Thorneley RNF. Mechanism of reaction of hydrogen peroxide with horseradish peroxidase:  Identification of intermediates in the catalytic cycle. J Am Chem Soc. 2001;123(48):11838-47. <https://doi.org/10.1021/ja011853>+

9. Liyanage PD, Weerathunge P, Singh M, Bansal V, Ramanathan R. L-Cysteine as an irreversible inhibitor of the peroxidase-mimic catalytic activity of 2-dimensional Ni-based nanozymes. Nanomaterials. 2021;11(5):1285. <https://doi.org/10.3390/nano11051285>

10. Karim MN, Singh M, Weerathunge P, Bian P, Zheng R, Dekiwadia C, et al. Visible-light-triggered reactive-oxygen-species-mediated antibacterial activity of peroxidase-mimic CuO nanorods. ACS Appl Nano Mater. 2018;1(4):1694-704. <https://doi.org/10.1021/acsanm.8b00153>

11. Hashmi S, Singh M, Weerathunge P, Mayes ELH, Mariathomas PD, N. Prasad S, et al. Cobalt sulfide nanosheets as peroxidase mimics for colorimetric detection of L-Cysteine. ACS Appl Nano Mater. 2021;4(12):13352-62. <https://doi.org/10.1021/acsanm.1c02851>

12. Bhattacharjee R, Tanaka S, Moriam S, Masud MK, Lin J, Alshehri SM, et al. Porous nanozymes: the peroxidase-mimetic activity of mesoporous iron oxide for the colorimetric and electrochemical detection of global DNA methylation. J Mater Chem B. 2018;6(29):4783-91. <https://doi.org/10.1039/C8TB01132J>

13. Ingenbosch KN, Quint S, Dyllick-Brenzinger M, Wunschik DS, Kiebist J, Süss P, et al. Singlet-oxygen generation by peroxidases and peroxygenases for chemoenzymatic synthesis. ChemBioChem. 2021;22(2):398-407. <https://doi.org/10.1002/cbic.202000326>

14. Kimura M, Umemoto Y, Kawano T. Hydrogen peroxide-independent generation of superoxide by plant peroxidase: hypotheses and supportive data employing ferrous ion as a model stimulus. Front Plant Sci. 2014;5:285. <https://doi.org/10.3389/fpls.2014.00285>

15. Plauck A, Stangland EE, Dumesic JA, Mavrikakis M. Active sites and mechanisms for H2O2 decomposition over Pd catalysts. PNAS. 2016;113(14):E1973-E82. <https://doi.org/10.1073/pnas.1602172113>

16. Zielonka J, Vasquez-Vivar J, Kalyanaraman B. Detection of 2-hydroxyethidium in cellular systems: a unique marker product of superoxide and hydroethidine. Nat Protoc. 2008;3(1):8-21. <https://doi.org/10.1038/nprot.2007.473>

17. Entradas T, Waldron S, Volk M. The detection sensitivity of commonly used singlet oxygen probes in aqueous environments. J Photochem Photobiol, B. 2020;204:111787. <https://doi.org/10.1016/j.jphotobiol.2020.111787>

18. Haber F, Weiss J. The catalytic decomposition of hydrogen peroxide by iron salts. Proc R Soc London, Ser A. 1934;147(861):332-51. <https://doi.org/10.1098/rspa.1934.0221>

19. Weiss J. The Free Radical Mechanism in the Reactions of Hydrogen peroxide. In: Frankenburg WG, Komarewsky VI, Rideal EK, editors. Adv Catal. 4: Academic Press; 1952. p. 343-65. <https://doi.org/10.1016/S0360-0564(08)60618-5>

20. Kitajima N, Fukuzumi S, Ono Y. Formation of superoxide ion during the decomposition of hydrogen peroxide on supported metal oxides. J Phys Chem. 1978;82(13):1505-9. <https://doi.org/10.1021/j100502a009>
